# Updating the knowledge of sand flies (Diptera, Psychodidae) in Rondônia State, Brazil

**DOI:** 10.3897/BDJ.10.e90015

**Published:** 2022-09-16

**Authors:** Antonio Marques Pereira Júnior, Moreno Magalhães de Souza Rodrigues, Jansen Fernandes Medeiros

**Affiliations:** 1 Fundação Oswaldo Cruz, Fiocruz Rondônia, Porto Velho, Brazil Fundação Oswaldo Cruz, Fiocruz Rondônia Porto Velho Brazil; 2 Instituto Nacional de Ciência e Tecnologia de Epidemiologia da Amazônia Ocidental, Porto Velho, Brazil Instituto Nacional de Ciência e Tecnologia de Epidemiologia da Amazônia Ocidental Porto Velho Brazil

**Keywords:** Phlebotominae, leishmaniasis, vectors, distribution, dataset

## Abstract

**Background:**

Sandflies are insects important for the transmission cycles of the leishmaniases. Despite being studied since the 1960s in the State of Rondônia (Brazil), several gaps exist regarding our working knowledge of these insects. This study aimed to construct an up-to-date database of sandflies using complementary information from the speciesLink database and the scientific literature, as well as to elaborate integrated abundance maps. We identified 153,155 records of sandflies captured in Rondônia between 1965-2021; after exclusion, 147,258 reports (speciesLink - 3,408, Rondônia studies – 143,850) associated with 15 genera and 140 species were mapped. The most abundant species observed were *Psychodopygusdavisi* (Root, 1934) (43,818 records), *Nyssomyiawhitmani* (Antunes & Coutinho, 1939) (12,594), *Psychodopyguscarrerai* (Barretto, 1946) (11,840), *Psychodopygushirsutus* (Mangabeira, 1942) (9,676), *Nyssomyiaantunesi* (Coutinho, 1939) (8,847), *Trichophoromyiaubiquitalis* (Mangabeira, 1942) (5,505), *Psychodopygusgeniculatus* (Mangabeira, 1941) (4,644), *Pintomyianevesi* (Damasceno & Arouck, 1956) (4,140), *Trichophoromyiaauraensis* (Mangabeira, 1942) (3,579), *Psychodopyguscomplexus* (Mangabeira, 1941) (2,659), *Nyssomyiafraihai* (Martins, Falcão & Silva, 1979) (2,504) and *Bichromomyiaflaviscutellata* (Mangabeira, 1942) (1,418). A total of 20 records of *Leishmania* detection corresponded to eight sand fly species. The present dataset provides updated information on the distribution of sandflies of Rondônia, including those considered potential vectors of *Leishmania*, which should prove useful to guide future studies.

**New information:**

The present study provides an extensive dataset built from all studies reporting phlebotomine sandflies in the Brazilian State of Rondônia. Online distribution maps can aid scientists who wish to consult the updated list of sand fly species and view the distribution of these insects, as well as those considered potential vectors of Leishmania. The results of the present study can serve as the basis for future studies on sandflies conducted in the State.

## Introduction

Sand flies are small dipteran insects mainly known for their role as vectors in the cycle of leishmaniasis transmission (Bates et al. 2015). In light of this importance, efforts to learn more about these insects have led to increasing amounts of data on the diversity, ecology, biology and genetics of these insects (Bates et al. 2015, Costa et al. 2021b). Sandflies are a diverse species found worldwide, with approximately 1,050 species described; Brazil is home to the greatest number of species, with 286 classifications registered (Galati 2021). Most of the knowledge gained has resulted from specialised training, which has greatly contributed to the diversity of the accumulated data.

Many regions in Brazil possess unexplored knowledge about these insects and it is highly possible that significant diversity remains to be discovered. Expanding the existing body of knowledge on these insects is important considering the role that sandflies play in the transmission of pathogens (Bates et al. 2015). Therefore, a robust dataset on the composition of these fauna could contribute to our understanding of main species in specific locales, as well as to potential vectors in a given region, constituting the first step for specific studies to identify the biological characteristics of regional species.

The Brazilian State of Rondônia, located in the Amazon Basin, borders the States of Acre, Amazonas and Mato Grosso and shares an international frontier with Bolivia. The State has the third highest incidence of cutaneous leishmaniasis (CL) in northern Brazil, with approximately 15,000 cases registered between 2007 and 2020. According to the Brazilian Ministry of Health, this incidence rate results from intense zoonotic transmission associated with human occupation in transmission foci (Teles et al. 2013, DATASUS 2020, Almeida et al. 2021). Previous studies have demonstrated that the following *Leishmania* species are responsible for human cases of CL in Rondônia: *Leishmaniaamazonensis* Lainson & Shaw, 1972, *Leishmaniabraziliensis* Vianna, 1911, *Leishmaniaguyanensis* Floch, 1954, *Leishmanialainsoni* Silveira, Ishikawa, Souza & Lainson, 1987, *Leishmanialindenbergi* Silveira, Ishikawa & Sousa, 2002, *Leishmanianaiffi* Lainson & Shaw, 1989 and *Leishmaniashawi* Lainson, Braga, Souza, Póvoa & Ishikawa, 1989 (Cantanhêde et al. 2015, Fagundes-Silva et al. 2015, Cantanhêde et al. 2019). However, despite the high prevalence of CL, knowledge on specific regional characteristics of the disease, including *Leishmania* species, reservoirs and vector species, remains incipient.

The first studies on sandflies were conducted in Rondônia in the 1960s (Martins et al. 1965). While some have been published in later decades, only from the 2000s has there been a substantial increase in accumulated data on sandflies (Gil et al. 2003, Gil et al. 2009, Teles et al. 2013, Galardo et al. 2015, Ogawa et al. 2016, Resadore et al. 2017, Resadore et al. 2018, Pereira Júnior et al. 2019b, Torchitte et al. 2020, Costa et al. 2021a, Silva et al. 2021). Nonetheless, studies describing vector distribution in the State are lacking. Accordingly, the present study aimed to build a database to provide an updated registry of sandflies and their potential vectors in Rondônia, in order to aid both specialists and non-specialists who work in the region.

## Materials and methods

### Study Area

Rondônia, located in the western part of the Amazon Basin, is third in terms of territorial extension amongst the States in the north of Brazil. The State covers an area of 237576 km^2^ and has a population of 1,815,278 inhabitants throughout its 52 municipalities; it shares borders with Bolivia and the neighbouring States of Acre, Amazonas and Mato Grosso. The local climate is tropical, with annual average temperatures varying between 19.2 and 26.6°C. The dominant vegetation is dense ombrophilous forest (Amazon tropical forest), yet Rondônia has experienced a decrease in vegetal cover over the last 40 years (Fearnside 2014). Moreover, an increasing incidence of CL cases has been associated with anthropogenic activities conducted in or near forest environments (Almeida et al. 2021b).

### Data distribution

We analysed data produced by zoological collections, such as the Phlebotomine Collection - Fiocruz/COLFLEB and Invertebrates Collection/INPA. These data are freely available on speciesLink (splink.cria.org.br/), a distributed information system that combines primary data from scientific collections. We also searched the scientific literature for all studies containing taxonomic and geographic information on sand fly species collected in Rondônia. The collected information consisted of total specimens and accompanying location coordinates in decimal format (long., lat.) to construct regional maps.

Distribution maps, constructed using QGIS3.4, revealed the most abundant species across the municipalities in Rondônia, with species abundance distributed in up to five classes: (i) 1-100 individuals, (ii) 101-500 individuals, (iii) 501-1000 individuals, (iv) 1001-2000 individuals and (v) > 2001 individuals. Data on *Leishmania* infection in each sand fly species were also compiled to build occurrence maps of potential vectors in the State. Finally, we constructed a web server with python applications containing information on the distribution of all species recorded throughout the State’s municipalities: https://sandfliesdb.herokuapp.com/.

## Checklists

### Updated list of sand flies from Rondônia State, Brazil

#### Bichromomyia flaviscutellata

(Mangabeira, 1942)

405CBE9C-A1DA-5866-9AC1-7AC6828815CA

https://sandfliesdb.herokuapp.com/

##### Distribution

Buritis, Cacaulândia, Cacoal, Campo Novo, Costa Marques, Guajará-Mirim, Itapuã do Oeste, Ji-Paraná, Machadinho d'Oeste, Monte Negro, Nova Mamoré, Porto Velho, São Francisco do Guaporé, Vale do Anari, Vilhena

##### Notes

Azevedo et al. 1993, Costa et al. 2021a, Galardo et al. 2015, Gil et al. 2003, Leão et al. 2020, Pereira Júnior et al. 2019a, Pereira Júnior et al. 2019b, Resadore et al. 2017, Resadore et al. 2018, Silva et al. 2021, Teles et al. 2013, Torchitte et al. 2020

#### Bichromomyia inornata

(Martins, Falcão & Silva, 1965)

F31F508C-25EA-531E-B1C3-7E6C43087DC7

https://sandfliesdb.herokuapp.com/

##### Distribution

Guajará-Mirim

##### Notes

Martins et al. 1965

#### Bichromomyia olmeca nociva

(Young & Arias, 1982)

3F3711BF-36CD-5513-804E-A6B8AE34F388

https://sandfliesdb.herokuapp.com/

##### Distribution

Cacaulândia, Costa Marques, Machadinho d'Oeste, Porto Velho

##### Notes

Costa et al. 2021a, Galardo et al. 2015, Pereira Júnior et al. 2019a, Pereira Júnior et al. 2019b, Resadore et al. 2018

#### Bichromomyia reducta

(Feliciangeli, Ramirez Pérez & Ramirez, 1988)

2CDC0806-F962-569E-ABF6-D24505610AA8

https://sandfliesdb.herokuapp.com/

##### Distribution

Porto Velho

##### Notes

Ogawa et al. 2016, Resadore et al. 2017, Silva et al. 2021

#### Brumptomyia avellari

(Costa Lima, 1932)

F4D95052-07CB-5475-B48E-656DEEADE047

https://sandfliesdb.herokuapp.com/

##### Distribution

Buritis, Cacaulândia, Campo Novo, Monte Negro

##### Notes

Gil et al. 2003

#### Brumptomyia brumpti

(Larrousse, 1920)

AD51BF8A-E102-55D5-91C8-C5E9BA663267

https://sandfliesdb.herokuapp.com/

##### Distribution

Cacaulândia, Cacoal, Costa Marques, Guajará-Mirim, Itapuã do Oeste, Ji-Paraná, Monte Negro, Nova Mamoré, Pimenta Bueno, Vale do Anari

##### Notes

Costa et al. 2021a, Leão et al. 2020, Pereira Júnior et al. 2019a, Pereira Júnior et al. 2019b, Teles et al. 2013, Torchitte et al. 2020

#### Brumptomyia cunhai

(Mangabeira, 1942)

05F53284-4445-52FC-BC47-1281EA092BC1

https://sandfliesdb.herokuapp.com/

##### Distribution

Cacaulândia

##### Notes

Gil et al. 2003

#### Brumptomyia mesai

Sherlock, 1962

7649AC44-A2C8-576F-8211-51EDD5AE2E53

https://sandfliesdb.herokuapp.com/

##### Distribution

Nova Mamoré, São Francisco do Guaporé

##### Notes

Costa et al. 2021a, Pereira Júnior et al. 2019a, Pereira Júnior et al. 2019b

#### Brumptomyia pentacantha

(Barretto, 1947)

8798CACA-6611-5517-BFAA-3DD3FD8D50F9

https://sandfliesdb.herokuapp.com/

##### Distribution

Cacaulândia, Guajará-Mirim, Monte Negro, Porto Velho

##### Notes

Gil et al. 2003, Martins et al. 1965, Silva et al. 2021, Teles et al. 2013

#### Brumptomyia pintoi

(Costa Lima, 1932)

4E6DFFBD-C814-567B-BC62-66E358532997

https://sandfliesdb.herokuapp.com/

##### Distribution

Buritis, Cacaulândia, Campo Novo, Costa Marques, Guajará-Mirim, Itapuã do Oeste, Monte Negro

##### Notes

Costa et al. 2021a, Gil et al. 2003, Martins et al. 1965, Pereira Júnior et al. 2019a, Pereira Júnior et al. 2019b

#### Brumptomyia travassosi

(Mangabeira, 1942)

30295CDF-D672-5342-9008-43B7CC247A21

https://sandfliesdb.herokuapp.com/

##### Distribution

Buritis, Cacaulândia, Campo Novo, Monte Negro, Porto Velho

##### Notes

Galardo et al. 2015, Gil et al. 2003

#### Evandromyia andersoni

(Le Pont & Desjeux, 1988)

2D24181C-1983-542C-82E9-62BF45E32C8B

https://sandfliesdb.herokuapp.com/

##### Distribution

Itapuã do Oeste

##### Notes

Leão et al. 2020

#### Evandromyia apurinan

Shimabukuro, Figueira & Silva, 2013

107CEB09-5954-574F-9AD5-544D3038B5BC

https://sandfliesdb.herokuapp.com/

##### Distribution

Itapuã do Oeste, Pimenta Bueno, Porto Velho

##### Notes

Costa et al. 2021a, Leão et al. 2020, Silva et al. 2021

#### Evandromyia bacula

(Martins, Falcão & Silva, 1965)

EF928838-E937-5685-85A4-45F5BCA1D765

https://sandfliesdb.herokuapp.com/

##### Distribution

Buritis, Cacaulândia, Campo Novo, Guajará-Mirim, Itapuã do Oeste, Monte Negro, Nova Mamoré, Pimenta Bueno, Porto Velho, Vale do Anari

##### Notes

Costa et al. 2021a, Galardo et al. 2015, Gil et al. 2003, Leão et al. 2020, Martins et al. 1965, Pereira Júnior et al. 2019b, Resadore et al. 2017, Silva et al. 2021, Teles et al. 2013

#### Evandromyia begonae

(Ortiz & Torrez, 1975)

011C5B19-3B8F-58AC-80D8-0317DE86050C

https://sandfliesdb.herokuapp.com/

##### Distribution

Cacaulândia, Porto Velho

##### Notes

Galardo et al. 2015, Gil et al. 2003

#### Evandromyia brachyphalla

(Mangabeira, 1941)

F87ABFBA-0B2D-59D5-AEC4-1CDB38FDA71D

https://sandfliesdb.herokuapp.com/

##### Distribution

Pimenta Bueno, Porto Velho

##### Notes

Galardo et al. 2015, Ogawa et al. 2016

#### Evandromyia carmelinoi

(Ryan, Fraiha, Lainson & Shaw, 1986)

ADCC3193-70F1-59FD-9630-F398F64FA17E

https://sandfliesdb.herokuapp.com/

##### Distribution

Pimenta Bueno

##### Notes

Costa et al. 2021a

#### Evandromyia evandroi

(Costa Lima & Antunes, 1936)

F1C6EB4F-9655-5223-A41B-7D1A20790FE9

https://sandfliesdb.herokuapp.com/

##### Distribution

Cacaulândia, Porto Velho

##### Notes

Galardo et al. 2015, Gil et al. 2003

#### Evandromyia georgii

(Freitas & Barrett, 2002)

54266204-6689-5A2B-AC15-E3BBD92C393A

https://sandfliesdb.herokuapp.com/

##### Distribution

Cacaulândia, Costa Marques, Guajará-Mirim, Itapuã do Oeste, Monte Negro, Pimenta Bueno, Porto Velho, São Francisco do Guaporé, Vale do Anari, Vilhena

##### Notes

Costa et al. 2021a, Ogawa et al. 2016, Pereira Júnior et al. 2019a, Pereira Júnior et al. 2019b, Resadore et al. 2017, Resadore et al. 2018, Silva et al. 2021

#### Evandromyia infraspinosa

(Mangabeira, 1941)

39639F5C-66F4-5430-A60B-07D308AB884B

https://sandfliesdb.herokuapp.com/

##### Distribution

Buritis, Cacaulândia, Campo Novo, Itapuã do Oeste, Monte Negro, Porto Velho

##### Notes

Galardo et al. 2015, Gil et al. 2003, Leão et al. 2020, Pereira Júnior et al. 2019a, Pereira Júnior et al. 2019b, Teles et al. 2013

#### Evandromyia inpai

(Young & Arias, 1977)

DDF14B5E-B40B-5D57-B65B-FA22841F120D

https://sandfliesdb.herokuapp.com/

##### Distribution

Cacaulândia

##### Notes

Gil et al. 2003

#### Evandromyia lenti

(Mangabeira, 1938)

BF1BAB37-8B43-5517-87F7-C846FC415F3E

https://sandfliesdb.herokuapp.com/

##### Distribution

Cacaulândia, Ji-Paraná, Monte Negro, Pimenta Bueno, Porto Velho

##### Notes

Costa et al. 2021a, Pereira Júnior et al. 2019a, Pereira Júnior et al. 2019b, Silva et al. 2021, Teles et al. 2013

#### Evandromyia monstruosa

(Floch & Abonnenc, 1944)

253037B3-695F-587B-8C76-45F5EDEBD432

https://sandfliesdb.herokuapp.com/

##### Distribution

Cacaulândia, Guajará-Mirim, Itapuã do Oeste, Monte Negro, Nova Mamoré, Porto Velho, Vale do Anari

#### Evandromyia pinottii

(Damasceno & Arouck, 1956)

94C6C12A-430C-58C2-83B4-2F656EF4EC04

https://sandfliesdb.herokuapp.com/

##### Distribution

Porto Velho

##### Notes

Galardo et al. 2015

#### Evandromyia piperiformis

Godoy, Cunha & Galati, 2017

8E69CC85-CFB1-551F-A09E-D33D9CA111E6

https://sandfliesdb.herokuapp.com/

##### Distribution

Cacaulândia, Costa Marques, Machadinho d'Oeste, Porto Velho, São Francisco do Guaporé

##### Notes

Costa et al. 2021a, Pereira Júnior et al. 2019a, Pereira Júnior et al. 2019b, Silva et al. 2021

#### Evandromyia saulensis

(Floch & Abonnenc, 1944)

249761AD-FE1B-5038-A1C5-7D27C99D99AB

https://sandfliesdb.herokuapp.com/

##### Distribution

Buritis, Cacaulândia, Campo Novo, Costa Marques, Guajará-Mirim, Itapuã do Oeste, Ji-Paraná, Monte Negro, Nova Mamoré, Pimenta Bueno, Porto Velho, Vale do Anari

##### Notes

Costa et al. 2021a, Galardo et al. 2015, Gil et al. 2003, Leão et al. 2020, Martins et al. 1965, Pereira Júnior et al. 2019a, Pereira Júnior et al. 2019b, Resadore et al. 2017, Resadore et al. 2018, Silva et al. 2021, Torchitte et al. 2020

#### Evandromyia sericea

(Floch & Abonnenc, 1944)

DD97DB37-ED5D-593E-86C6-A7ADE1A8D82E

https://sandfliesdb.herokuapp.com/

##### Distribution

Cacaulândia, Itapuã do Oeste, Porto Velho

##### Notes

Gil et al. 2003, Leão et al. 2020, Silva et al. 2021

#### Evandromyia sp. de Baduel

(Floch & Abonnenc, 1945)

C9BE2C29-8DB5-53D6-9B5E-621AD761137A

https://sandfliesdb.herokuapp.com/

##### Distribution

Guajará-Mirim

##### Notes

Martins et al. 1965

#### Evandromyia tarapacaensis

(Le Pont, Torrez-Espejo & Galati, 1997)

DC10DFD5-5F66-51F9-878C-0F6BD335DDBE

https://sandfliesdb.herokuapp.com/

##### Distribution

Itapuã do Oeste, Monte Negro, Nova Mamoré, Porto Velho

##### Notes

Leão et al. 2020, Pereira Júnior et al. 2019a, Pereira Júnior et al. 2019b, Resadore et al. 2017, Resadore et al. 2018, Silva et al. 2021, Teles et al. 2013

#### Evandromyia termitophila

(Martins, Falcão & Silva, 1964)

347DDD1E-8092-5FA7-92EA-763EF8BF98AE

https://sandfliesdb.herokuapp.com/

##### Distribution

Buritis, Cacaulândia, Campo Novo, Guajará-Mirim, Itapuã do Oeste, Monte Negro, Nova Mamoré, Porto Velho, Vale do Anari

##### Notes

Gil et al. 2003, Leão et al. 2020, Martins et al. 1965, Pereira Júnior et al. 2019a, Pereira Júnior et al. 2019b, Resadore et al. 2017, Resadore et al. 2018, Silva et al. 2021, Teles et al. 2016

#### Evandromyia walkeri

(Newstead, 1914)

9826991D-83CB-59A8-BEE1-332CC8E435DB

https://sandfliesdb.herokuapp.com/

##### Distribution

Cacaulândia, Cacoal, Costa Marques, Guajará-Mirim, Itapuã do Oeste, Ji-Paraná, Machadinho d'Oeste, Monte Negro, Nova Mamoré, Pimenta Bueno, Porto Velho, São Francisco do Guaporé, Vale do Anari

##### Notes

Costa et al. 2021a, Galardo et al. 2015, Leão et al. 2020, Martins et al. 1965, Pereira Júnior et al. 2019a, Pereira Júnior et al. 2019b, Resadore et al. 2017, Resadore et al. 2018, Teles et al. 2013, Torchitte et al. 2020

#### Evandromyia williamsi

(Damasceno, Causey & Arouck, 1945)

4B65FF2C-9A40-5C4F-B0B4-BFB72C5C0BDF

https://sandfliesdb.herokuapp.com/

##### Distribution

Cacaulândia, Costa Marques, Itapuã do Oeste, Monte Negro, Nova Mamoré, Porto Velho

##### Notes

Costa et al. 2021a, Galardo et al. 2015, Gil et al. 2003, Leão et al. 2020, Pereira Júnior et al. 2019a, Pereira Júnior et al. 2019b, Resadore et al. 2018, Silva et al. 2021, Teles et al. 2013

#### Evandromyia wilsoni

(Damasceno & Causey, 1945)

B9CA8167-B897-510D-AAB8-40C3CCBF4EAE

https://sandfliesdb.herokuapp.com/

##### Distribution

Buritis, Cacaulândia, Campo Novo, Costa Marques, Guajará-Mirim, Itapuã do Oeste, Machadinho d'Oeste, Monte Negro, Nova Mamoré, Pimenta Bueno, Porto Velho

##### Notes

Costa et al. 2021a, Gil et al. 2003, Leão et al. 2020, Martins et al. 1965, Pereira Júnior et al. 2019a, Pereira Júnior et al. 2019b, Resadore et al. 2017, Resadore et al. 2018, Silva et al. 2021, Teles et al. 2013

#### Lutzomyia caligata

Martins, Falcão & Silva, 1965

E3E5BD45-7939-5214-A4B1-F173B28A8503

https://sandfliesdb.herokuapp.com/

##### Distribution

Guajará-Mirim

##### Notes

Martins et al. 1965

#### Lutzomyia carvalhoi

(Damasceno, Causey & Arouck, 1945)

4AC37A42-7398-545B-A47A-AC100D42DD7C

https://sandfliesdb.herokuapp.com/

##### Distribution

Buritis, Cacaulândia, Campo Novo, Monte Negro, Porto Velho

##### Notes

Galardo et al. 2015, Gil et al. 2003

#### Lutzomyia evangelistai

Martins & Fraiha, 1971

7167478B-A781-5C2F-81F2-4015D5BEFFCE

https://sandfliesdb.herokuapp.com/

##### Distribution

Buritis, Cacaulândia, Campo Novo, Itapuã do Oeste, Monte Negro, Porto Velho, Vale do Anari

##### Notes

Azevedo et al. 1993, Gil et al. 2003, Pereira Júnior et al. 2019a, Pereira Júnior et al. 2019b, Resadore et al. 2017, Silva et al. 2021, Teles et al. 2013

#### Lutzomyia falcata

Young, Morales & Ferro, 1994

2D926CF1-0ED7-5D16-8EAE-3F5A67FF3FE2

https://sandfliesdb.herokuapp.com/

##### Distribution

Porto Velho

##### Notes

Resadore et al. 2017

#### Lutzomyia flabellata

Martins & Silva, 1964

8DA6563F-EE2E-5EB5-AB5C-D05DFFB87119

https://sandfliesdb.herokuapp.com/

##### Distribution

Itapuã do Oeste, Vale do Anari

##### Notes

Leão et al. 2020, Pereira Júnior et al. 2019a, Pereira Júnior et al. 2019b, Resadore et al. 2018

#### Lutzomyia gomezi

(Nitzulescu, 1931)

EAE2D441-9595-5E85-B331-FED66B6AD970

https://sandfliesdb.herokuapp.com/

##### Distribution

Buritis, Cacaulândia, Campo Novo, Guajará-Mirim, Monte Negro, Porto Velho

##### Notes

Azevedo et al. 1993, Gil et al. 2003, Martins et al. 1965, Silva et al. 2021

#### Lutzomyia longipalpis

(Lutz & Neiva, 1912)

1A4D96D2-12BD-5B0B-8E38-CDD8D7732C29

https://sandfliesdb.herokuapp.com/

##### Distribution

Cacaulândia, Monte Negro

##### Notes

Gil et al. 2003, Teles et al. 2013

#### Lutzomyia marinkellei

Young, 1979

0183103C-3963-57A5-BCD4-D5B6B77106DC

https://sandfliesdb.herokuapp.com/

##### Distribution

Itapuã do Oeste

##### Notes

Pereira Júnior et al. 2019a, Pereira Júnior et al. 2019b

#### Lutzomyia sherlocki

Martins, Silva & Falcão, 1971

7A32D78F-8CFB-5655-870C-26CF681079E0

https://sandfliesdb.herokuapp.com/

##### Distribution

Cacaulândia, Cacoal, Costa Marques, Itapuã do Oeste, Ji-Paraná, Machadinho d'Oeste, Monte Negro, Nova Mamoré, Pimenta Bueno, Porto Velho, São Francisco do Guaporé, Vale do Anari, Vilhena

##### Notes

Costa et al. 2021a, Leão et al. 2020, Pereira Júnior et al. 2019a, Pereira Júnior et al. 2019b, Resadore et al. 2017, Resadore et al. 2018, Silva et al. 2021, Teles et al. 2013, Torchitte et al. 2020

#### Martinsmyia waltoni

(Arias, Freitas & Barrett, 1984)

65A3FB73-FA3B-51A0-B5E0-D56AFE0EA74C

https://sandfliesdb.herokuapp.com/

##### Distribution

Buritis, Cacaulândia, Campo Novo, Monte Negro

##### Notes

Gil et al. 2003, Pereira Júnior et al. 2019a, Pereira Júnior et al. 2019b

#### Micropygomyia acanthopharynx

(Martins, Falcão & Silva, 1962)

36E5095B-7AD1-5E3E-B9B2-1E038BC4AC2E

https://sandfliesdb.herokuapp.com/

##### Distribution

Cacaulândia, Monte Negro

##### Notes

Pereira Júnior et al. 2019a, Pereira Júnior et al. 2019b, Teles et al. 2013

#### Micropygomyia cayennensis cayennensis

(Floch & Abonnenc, 1941)

B2AE6954-9345-53A9-BB1D-CFAB7588F7BF

https://sandfliesdb.herokuapp.com/

##### Distribution

Cacaulândia

##### Notes

Gil et al. 2003, Castellón Bermúdez 2009

#### Micropygomyia echinatopharynx

Andrade Filho, Galati, Andrade & Facão, 2004

5E6F56D1-9D38-59E2-B207-F3056E13EAE4

https://sandfliesdb.herokuapp.com/

##### Distribution

Costa Marques

##### Notes

Costa et al. 2021a

#### Micropygomyia longipennis

(Barretto, 1946)

E651C9FC-6D8E-5A36-81D3-613AA8ED445D

https://sandfliesdb.herokuapp.com/

##### Distribution

Buritis, Cacaulândia, Campo Novo, Itapuã do Oeste, Monte Negro, Porto Velho

##### Notes

Galardo et al. 2015, Gil et al. 2003, Resadore et al. 2018

#### Micropygomyia micropyga

(Mangabeira, 1942)

792DEADF-E9BA-5AF7-BAFE-F01383709D89

https://sandfliesdb.herokuapp.com/

##### Distribution

Cacaulândia, Guajará-Mirim, Itapuã do Oeste, Monte Negro, Porto Velho

##### Notes

Galardo et al. 2015, Gil et al. 2003, Martins et al. 1965, Resadore et al. 2018, Teles et al. 2013

#### Micropygomyia oswaldoi

(Mangabeira, 1942)

B3047ACA-F29A-5B13-8BE9-B0F5CC62F8B9

https://sandfliesdb.herokuapp.com/

##### Distribution

Cacaulândia, Porto Velho

##### Notes

Galardo et al. 2015, Gil et al. 2003

#### Micropygomyia peresi

(Mangabeira, 1942)

13C609E3-F325-587A-A873-F53FD1F3FE11

https://sandfliesdb.herokuapp.com/

##### Distribution

Cacaulândia, Itapuã do Oeste

##### Notes

Gil et al. 2003, Leão et al. 2020

#### Micropygomyia pilosa

(Damasceno & Causey, 1944)

F0917988-8FCA-5C15-904C-E45477F8D082

https://sandfliesdb.herokuapp.com/

##### Distribution

Buritis, Cacaulândia, Campo Novo, Monte Negro, Porto Velho

##### Notes

Gil et al. 2003, Ogawa et al. 2016, Pereira Júnior et al. 2019a, Pereira Júnior et al. 2019b

#### Micropygomyia rorotaensis

(Floch & Abonnenc, 1944)

1AEB4FF5-4028-5B99-9288-B305C244509A

https://sandfliesdb.herokuapp.com/

##### Distribution

Buritis, Cacaulândia, Campo Novo, Costa Marques, Itapuã do Oeste, Ji-Paraná, Machadinho d'Oeste, Monte Negro, Porto Velho, Vale do Anari

##### Notes

Costa et al. 2021a, Galardo et al. 2015, Gil et al. 2003, Leão et al. 2020, Pereira Júnior et al. 2019a, Pereira Júnior et al. 2019b, Resadore et al. 2017, Resadore et al. 2018, Silva et al. 2021, Torchitte et al. 2020

#### Micropygomyia trinidadensis

(Newstead, 1922)

3A93E3B9-ED15-5F84-86DD-16FC0F2F8FB4

https://sandfliesdb.herokuapp.com/

##### Distribution

Buritis, Cacaulândia, Campo Novo, Costa Marques, Guajará-Mirim, Itapuã do Oeste, Monte Negro, Porto Velho

##### Notes

Costa et al. 2021a, Galardo et al. 2015, Gil et al. 2003, Martins et al. 1965, Pereira Júnior et al. 2019a, Pereira Júnior et al. 2019b, Resadore et al. 2018

#### Micropygomyia villelai

(Mangabeira, 1942)

036BBD1F-6C23-5123-B427-D74744470C19

https://sandfliesdb.herokuapp.com/

##### Distribution

Buritis, Cacaulândia, Cacoal, Campo Novo, Itapuã do Oeste, Ji-Paraná, Machadinho d'Oeste, Monte Negro, Porto Velho

##### Notes

Costa et al. 2021b, Gil et al. 2003, Leão et al. 2020, Pereira Júnior et al. 2019a, Pereira Júnior et al. 2019b, Silva et al. 2021, Teles et al. 2013, Torchitte et al. 2020

#### Migonemyia cerqueirai

(Causey & Damasceno, 1945)

17F6E812-1715-5115-825D-83C85DDAA88F

https://sandfliesdb.herokuapp.com/

##### Distribution

Guajará-Mirim

##### Notes

Martins et al. 1965

#### Migonemyia migonei

(França, 1920)

49033A29-A793-5BDB-92B4-5E170F0D1F0B

https://sandfliesdb.herokuapp.com/

##### Distribution

Buritis, Cacaulândia, Cacoal, Campo Novo, Guajará-Mirim, Itapuã do Oeste, Ji-Paraná, Monte Negro, Nova Mamoré, Porto Velho

##### Notes

Galardo et al. 2015, Gil et al. 2003, Leão et al. 2020, Pereira Júnior et al. 2019a, Pereira Júnior et al. 2019b, Resadore et al. 2018, Silva et al. 2021, Torchitte et al. 2020

#### Nyssomyia anduzei

(Rozeboom, 1942)

91D83CF8-ACAB-5CAF-87D8-8DB4408A301C

https://sandfliesdb.herokuapp.com/

##### Distribution

Buritis, Cacaulândia, Cacoal, Campo Novo, Guajará-Mirim, Itapuã do Oeste, Monte Negro, Nova Mamoré, Pimenta Bueno, Porto Velho, Vale do Anari

##### Notes

Azevedo et al. 1993, Galardo et al. 2015, Gil et al. 2003, Leão et al. 2020, Martins et al. 1965, Ogawa et al. 2016, Pereira Júnior et al. 2019a, Pereira Júnior et al. 2019b, Resadore et al. 2017, Resadore et al. 2018

#### Nyssomyia antunesi

(Coutinho, 1939)

0BBE5BCC-2392-55B7-9E07-DC3F2E333E05

https://sandfliesdb.herokuapp.com/

##### Distribution

Buritis, Cacaulândia, Cacoal, Campo Novo, Costa Marques, Guajará-Mirim, Itapuã do Oeste, Ji-Paraná, Machadinho d'Oeste, Monte Negro, Nova Mamoré, Pimenta Bueno, Porto Velho, São Francisco do Guaporé, Vale do Anari, Vilhena

##### Notes

Costa et al. 2021a, Galardo et al. 2015, Gil et al. 2003, Leão et al. 2020, Martins et al. 1965, Ogawa et al. 2016, Pereira Júnior et al. 2019a, Pereira Júnior et al. 2019b, Resadore et al. 2017, Resadore et al. 2018, Silva et al. 2021, Teles et al. 2013, Torchitte et al. 2020

#### Nyssomyia delsionatali

Galati & Galvis, 2012

FB747CCA-ACE2-5DE5-B30C-BB760BB220BB

https://sandfliesdb.herokuapp.com/

##### Distribution

Cacoal, Costa Marques, Itapuã do Oeste, Ji-Paraná, Machadinho d'Oeste, Pimenta Bueno, Porto Velho, Vale do Anari, Vilhena

##### Notes

Costa et al. 2021a, Pereira Júnior et al. 2019a, Pereira Júnior et al. 2019b, Silva et al. 2021

#### Nyssomyia fraihai

(Martins, Falcão & Silva, 1979)

8099CBC4-F1FC-5AEF-8009-32BA2D4982C6

https://sandfliesdb.herokuapp.com/

##### Distribution

Itapuã do Oeste, Machadinho d'Oeste, Nova Mamoré, Pimenta Bueno, Porto Velho, Vale do Anari, Vilhena

##### Notes

Azevedo et al. 1993, Costa et al. 2021b, Galardo et al. 2015, Gil et al. 2003, Leão et al. 2020, Ogawa et al. 2016, Pereira Júnior et al. 2019a, Pereira Júnior et al. 2019b, Resadore et al. 2017, Resadore et al. 2018, Silva et al. 2021

#### Nyssomyia richardwardi

(Ready & Fraiha, 1981)

694D18F4-9382-5007-A92F-CDF3ECAE0F91

https://sandfliesdb.herokuapp.com/

##### Distribution

Cacaulândia, Cacoal, Itapuã do Oeste, Ji-Paraná, Machadinho d'Oeste, Monte Negro, Nova Mamoré, Porto Velho, Vale do Anari, Vilhena

##### Notes

Azevedo et al. 1993, Costa et al. 2021b, Gil et al. 2003, Leão et al. 2020, Pereira Júnior et al. 2019a, Pereira Júnior et al. 2019b, Resadore et al. 2018, Silva et al. 2021

#### Nyssomyia shawi

(Fraiha, Ward & Ready, 1981)

BFB97742-682A-5DF5-8DE7-02DD508F62BC

https://sandfliesdb.herokuapp.com/

##### Distribution

Buritis, Cacaulândia, Campo Novo, Itapuã do Oeste, Monte Negro, Nova Mamoré, Pimenta Bueno, Porto Velho

##### Notes

Azevedo et al. 1993, Galardo et al. 2015, Gil et al. 2003, Leão et al. 2020, Ogawa et al. 2016, Pereira Júnior et al. 2019a, Pereira Júnior et al. 2019b, Resadore et al. 2018, Silva et al. 2021, Teles et al. 2013

#### Nyssomyia umbratilis

(Ward & Fraiha, 1977)

32859C7F-09F0-5910-BF61-480AC1A588D9

https://sandfliesdb.herokuapp.com/

##### Distribution

Buritis, Cacaulândia, Campo Novo, Guajará-Mirim, Itapuã do Oeste, Machadinho d'Oeste, Monte Negro, Nova Mamoré, Pimenta Bueno, Porto Velho, Vale do Anari, Vilhena

##### Notes

Azevedo et al. 1993, Costa et al. 2021a, Galardo et al. 2015, Gil et al. 2003, Leão et al. 2020, Ogawa et al. 2016, Pereira Júnior et al. 2019a, Pereira Júnior et al. 2019b, Resadore et al. 2017, Resadore et al. 2018, Silva et al. 2021, Teles et al. 2013

#### Nyssomyia urbinattii

Galati & Galvis, 2012

322A9682-FE2D-5653-8FC3-F716FB62C5BE

https://sandfliesdb.herokuapp.com/

##### Distribution

Ji-Paraná, Machadinho d'Oeste, Pimenta Bueno, São Francisco do Guaporé

##### Notes

Costa et al. 2021a, Torchitte et al. 2020

#### Nyssomyia whitmani

(Antunes & Coutinho, 1939)

8DD1092A-A7A0-5E5D-AE30-679FF845F7D9

https://sandfliesdb.herokuapp.com/

##### Distribution

Buritis, Cacaulândia, Cacoal, Campo Novo, Costa Marques, Guajará-Mirim, Itapuã do Oeste, Ji-Paraná, Machadinho d'Oeste, Monte Negro, Nova Mamoré, Pimenta Bueno, Porto Velho, São Francisco do Guaporé, Vale do Anari, Vilhena

##### Notes

Costa et al. 2021a, Gil et al. 2003, Leão et al. 2020, Ogawa et al. 2016, Pereira Júnior et al. 2019a, Pereira Júnior et al. 2019b, Resadore et al. 2017, Resadore et al. 2018, Silva et al. 2021, Teles et al. 2013, Torchitte et al. 2020

#### Nyssomyia yuilli pajoti

(Abonnenc, Léger& Fauran 1979)

1741F56A-5BC9-58ED-AF7C-573C2BEF18A1

https://sandfliesdb.herokuapp.com/

##### Distribution

Porto Velho

##### Notes

Galardo et al. 2015

#### Nyssomyia yuilli yuilli

(Young & Porter, 1972)

52A56F22-60F2-5778-A8CE-35AB724A1408

https://sandfliesdb.herokuapp.com/

##### Distribution

Cacaulândia, Guajará-Mirim, Porto Velho

##### Notes

Azevedo et al. 1993, Galardo et al. 2015, Gil et al. 2003

#### Pintomyia damascenoi

(Mangabeira, 1941)

2CD8696A-29BD-5E5E-AA93-798EF6D59B32

https://sandfliesdb.herokuapp.com/

##### Distribution

Buritis, Cacaulândia, Campo Novo, Monte Negro, Porto Velho

##### Notes

Galardo et al. 2015, Gil et al. 2003

#### Pintomyia duckei

Oliveira, Alencar & Freitas, 2018

B5783033-9E37-5E9F-90F1-F80CEB5B732B

https://sandfliesdb.herokuapp.com/

##### Distribution

Itapuã do Oeste

##### Notes

Leão et al. 2020

#### Pintomyia fiocruzi

Pereira-Júnior, Pessoa, Marialva & Medeiros, 2019

65381241-87D2-525C-B2F2-67B6A4BC80BB

https://sandfliesdb.herokuapp.com/

##### Distribution

Costa Marques, Itapuã do Oeste, Machadinho d'Oeste, Nova Mamoré, Porto Velho

##### Notes

Costa et al. 2021a, Leão et al. 2020, Pereira Júnior et al. 2019a, Pereira Júnior et al. 2019b, Silva et al. 2021

#### Pintomyia gruta

(Ryan, 1986)

927820B0-8AEE-5ADE-9B15-3E089ABF84C3

https://sandfliesdb.herokuapp.com/

##### Distribution

Porto Velho

##### Notes

Galardo et al. 2015, Ogawa et al. 2016

#### Pintomyia nevesi

(Damasceno & Arouck, 1956)

01949AE5-3574-58FA-9D90-863FABBB2C2C

https://sandfliesdb.herokuapp.com/

##### Distribution

Buritis, Cacaulândia, Cacoal, Campo Novo, Costa Marques, Guajará-Mirim, Itapuã do Oeste, Ji-Paraná, Monte Negro, Nova Mamoré, Porto Velho, São Francisco do Guaporé, Vale do Anari, Vilhena

##### Notes

Costa et al. 2021a, Gil et al. 2003, Martins et al. 1965, Pereira Júnior et al. 2019a, Pereira Júnior et al. 2019b, Resadore et al. 2018, Silva et al. 2021, Teles et al. 2013, Torchitte et al. 2020

#### Pintomyia odax

(Fairchild & Hertig, 1961)

195B4C37-A5ED-5D87-9847-29B841775AEC

https://sandfliesdb.herokuapp.com/

##### Distribution

Guajará-Mirim, Porto Velho

##### Notes

Biancardi et al. 1982

#### Pintomyia pacae

(Floch & Abonnenc, 1943)

47B27F2A-0048-50D5-80CE-99196192855A

https://sandfliesdb.herokuapp.com/

##### Distribution

Cacaulândia

##### Notes

Gil et al. 2003

#### Pintomyia serrana

(Damasceno & Arouck, 1949)

404D032E-87E6-5AA3-B22E-1F9174158B6F

https://sandfliesdb.herokuapp.com/

##### Distribution

Buritis, Cacaulândia, Cacoal, Campo Novo, Costa Marques, Guajará-Mirim, Itapuã do Oeste, Ji-Paraná, Monte Negro, Nova Mamoré, Porto Velho, Vale do Anari

##### Notes

Costa et al. 2021a, Gil et al. 2003, Leão et al. 2020, Martins et al. 1965, Pereira Júnior et al. 2019b, Pereira Júnior et al. 2015, Silva et al. 2021, Teles et al. 2013, Torchitte et al. 2020

#### Pressatia calcarata

(Martins & Silva, 1964)

7FA98A32-7C9F-56D5-AEC4-1D614EE1BEF2

https://sandfliesdb.herokuapp.com/

##### Distribution

Costa Marques, Guajará-Mirim, Itapuã do Oeste, Nova Mamoré

##### Notes

Costa et al. 2021a, Leão et al. 2020, Martins et al. 1965, Pereira Júnior et al. 2019a, Pereira Júnior et al. 2019b

#### Pressatia choti

(Floch & Abonnenc, 1941)

EAB998CD-D880-51EE-88EE-41707C986A4E

https://sandfliesdb.herokuapp.com/

##### Distribution

Porto Velho

##### Notes

Galardo et al. 2015

#### Pressatia triacantha

(Mangabeira, 1942)

05B928CD-85DB-5B73-9E4E-BB79258D0D67

https://sandfliesdb.herokuapp.com/

##### Distribution

Buritis, Cacaulândia, Campo Novo, Guajará-Mirim, Itapuã do Oeste, Monte Negro, Nova Mamoré, Porto Velho

##### Notes

Galardo et al. 2015, Gil et al. 2003, Leão et al. 2020, Martins et al. 1965, Pereira Júnior et al. 2019a, Pereira Júnior et al. 2019b, Silva et al. 2021, Teles et al. 2013

#### Pressatia trispinosa

(Mangabeira, 1942)

E8459618-B20A-595C-801B-0E1F4341DB9C

https://sandfliesdb.herokuapp.com/

##### Distribution

Cacaulândia, Costa Marques

##### Notes

Costa et al. 2021a, Gil et al. 2003

#### Psathyromyia abonnenci

(Floch & Chassignet, 1947)

CD1B8D87-0B45-5711-BC10-864E0FA73D45

https://sandfliesdb.herokuapp.com/

##### Distribution

Buritis, Cacaulândia, Campo Novo, Itapuã do Oeste, Monte Negro, Porto Velho

##### Notes

Gil et al. 2003, Leão et al. 2020, Martins et al. 1965, Resadore et al. 2018

#### Psathyromyia abunaensis

(Martins, Falcão & Silva, 1965)

C352C277-7D76-5793-9B07-881FF6F546CC

https://sandfliesdb.herokuapp.com/

##### Distribution

Cacaulândia, Guajará-Mirim, Monte Negro, Pimenta Bueno, Porto Velho

##### Notes

Costa et al. 2021b, Gil et al. 2003, Martins et al. 1965, Ogawa et al. 2016, Silva et al. 2021, Teles et al. 2013

#### Psathyromyia aragaoi

(Costa Lima, 1932)

A997D1E3-CD08-57A9-B9E0-E39EC4AEAB63

https://sandfliesdb.herokuapp.com/

##### Distribution

Buritis, Cacaulândia, Campo Novo, Costa Marques, Guajará-Mirim, Itapuã do Oeste, Monte Negro, Nova Mamoré, Porto Velho, São Francisco do Guaporé, Vale do Anari, Vilhena

##### Notes

Galardo et al. 2015, Gil et al. 2003, Leão et al. 2020, Martins et al. 1965, Pereira Júnior et al. 2019a, Pereira Júnior et al. 2019b, Resadore et al. 2017, Resadore et al. 2018, Silva et al. 2021, Teles et al. 2013

#### Psathyromyia barrettoi barretoi

(Mangabeira, 1942)

E4061591-0596-5BF6-8493-252E33DBF4B0

https://sandfliesdb.herokuapp.com/

##### Distribution

Buritis, Cacaulândia, Campo Novo, Itapuã do Oeste, Monte Negro, Porto Velho

##### Notes

Gil et al. 2003, Pereira Júnior et al. 2019a, Pereira Júnior et al. 2019b, Silva et al. 2021, Teles et al. 2013

#### Psathyromyia bigeniculata

(Floch & Abonnenc, 1941)

1B10823E-A073-5A07-A3FF-545F2E10565D

https://sandfliesdb.herokuapp.com/

##### Distribution

Buritis, Cacaulândia, Campo Novo, Costa Marques, Guajará-Mirim, Itapuã do Oeste, Machadinho d'Oeste, Monte Negro, Porto Velho

##### Notes

Azevedo et al. 1993, Costa et al. 2021b, Gil et al. 2003, Leão et al. 2020, Martins et al. 1965, Pereira Júnior et al. 2015, Silva et al. 2021, Teles et al. 2013

#### Psathyromyia brasiliensis

(Costa Lima, 1932)

630D33DD-F7D3-5139-BF5C-B92CBC2A033F

https://sandfliesdb.herokuapp.com/

##### Distribution

Cacaulândia, Porto Velho

##### Notes

Azevedo et al. 1993, Galardo et al. 2015, Gil et al. 2003, Resadore et al. 2017

#### Psathyromyia campbelli

(Damasceno, Causey & Arouck, 1945)

BEAABE13-EFB0-5B19-9381-253AA11D40F6

https://sandfliesdb.herokuapp.com/

##### Distribution

Buritis, Cacaulândia, Campo Novo, Costa Marques, Itapuã do Oeste, Ji-Paraná, Monte Negro, Nova Mamoré

##### Notes

Costa et al. 2021a, Gil et al. 2003, Leão et al. 2020, Pereira Júnior et al. 2019a, Pereira Júnior et al. 2019b, Teles et al. 2013, Torchitte et al. 2020

#### Psathyromyia coutinhoi

(Mangabeira, 1942)

36978D8F-874F-5EFF-A755-2F809F0E1DC7

https://sandfliesdb.herokuapp.com/

##### Distribution

Guajará-Mirim, Itapuã do Oeste, Monte Negro, Porto Velho

##### Notes

Martins et al. 1965, Pereira Júnior et al. 2019a, Pereira Júnior et al. 2019b, Silva et al. 2021, Teles et al. 2013

#### Psathyromyia dasymera

(Fairchild & Hertig, 1961)

FC7B7FB4-49A0-5D86-A92B-FA12595032CA

https://sandfliesdb.herokuapp.com/

##### Distribution

Porto Velho

##### Notes

Biancardi et al. 1982

#### Psathyromyia dendrophyla

(Mangabeira, 1942)

25CD9930-5B71-5E6B-B20D-26AAFBF14419

https://sandfliesdb.herokuapp.com/

##### Distribution

Buritis, Cacaulândia, Cacoal, Campo Novo, Costa Marques, Guajará-Mirim, Itapuã do Oeste, Ji-Paraná, Monte Negro, Nova Mamoré, Pimenta Bueno, Porto Velho, Vale do Anari

##### Notes

Azevedo et al. 1993, Costa et al. 2021a, Galardo et al. 2015, Gil et al. 2003, Leão et al. 2020, Martins et al. 1965, Pereira Júnior et al. 2019a, Pereira Júnior et al. 2019b, Resadore et al. 2017, Resadore et al. 2018, Silva et al. 2021, Teles et al. 2013, Torchitte et al. 2020

#### Psathyromyia dreisbachi

(Causey & Damasceno, 1945)

1B22555E-302A-53A8-8DCE-88A4ADB5D194

https://sandfliesdb.herokuapp.com/

##### Distribution

Cacaulândia, Cacoal, Guajará-Mirim, Itapuã do Oeste, Ji-Paraná, Monte Negro, Nova Mamoré, Porto Velho

##### Notes

Galardo et al. 2015, Gil et al. 2003, Leão et al. 2020, Martins et al. 1965, Pereira Júnior et al. 2019a, Pereira Júnior et al. 2019b, Silva et al. 2021

#### Psathyromyia elizabethdorvalae

Brilhante, Sábio & Galati, 2017

4BC9C80D-F57E-5C96-B5A6-55E13ABB3E39

https://sandfliesdb.herokuapp.com/

##### Distribution

Costa Marques, Itapuã do Oeste, Machadinho d'Oeste, Monte Negro, Nova Mamoré, Porto Velho, São Francisco do Guaporé

##### Notes

Costa et al. 2021a, Pereira Júnior et al. 2019b, Pereira Júnior et al. 2015, Silva et al. 2021

#### Psathyromyia hermanlenti

(Martins, Silva & Falcão, 1970)

6C5908A9-96EF-5F67-95C5-5DEEFC9C0715

https://sandfliesdb.herokuapp.com/

##### Distribution

Cacaulândia, Cacoal, Costa Marques, Guajará-Mirim, Ji-Paraná, Machadinho d'Oeste, Monte Negro, Nova Mamoré, Pimenta Bueno, Porto Velho, São Francisco do Guaporé, Vilhena

##### Notes

Costa et al. 2021a, Pereira Júnior et al. 2019a, Pereira Júnior et al. 2019b, Resadore et al. 2017, Resadore et al. 2018, Silva et al. 2021, Torchitte et al. 2020

#### Psathyromyia inflata

(Floch & Abonnenc, 1944)

2FF7CA1F-E23D-5821-8B00-F9CA88434BF7

https://sandfliesdb.herokuapp.com/

##### Distribution

Porto Velho

##### Notes

Galardo et al. 2015

#### Psathyromyia lutziana

(Costa Lima, 1932)

61AD5EB3-8993-57DB-819B-89CBD216550B

https://sandfliesdb.herokuapp.com/

##### Distribution

Buritis, Cacaulândia, Campo Novo, Guajará-Mirim, Itapuã do Oeste, Ji-Paraná, Machadinho d'Oeste, Monte Negro, Nova Mamoré, Pimenta Bueno, Porto Velho, São Francisco do Guaporé, Vale do Anari

##### Notes

Costa et al. 2021a, Galardo et al. 2015, Gil et al. 2003, Leão et al. 2020, Martins et al. 1965, Ogawa et al. 2016, Pereira Júnior et al. 2019a, Pereira Júnior et al. 2019b, Resadore et al. 2017, Resadore et al. 2018, Silva et al. 2021, Teles et al. 2013, Torchitte et al. 2020

#### Psathyromyia pradobarrientosi

(Le Pont, Matias, Martinez & Dujardin, 2004)

68963436-2696-5950-89E5-B684970076D4

https://sandfliesdb.herokuapp.com/

##### Distribution

Cacoal, Guajará-Mirim, Ji-Paraná, Machadinho d'Oeste, Monte Negro, Nova Mamoré, Pimenta Bueno, Porto Velho, São Francisco do Guaporé, Vilhena

##### Notes

Costa et al. 2021a, Martins et al. 1965, Pereira Júnior et al. 2019a, Pereira Júnior et al. 2019b, Resadore et al. 2017, Silva et al. 2021, Torchitte et al. 2020

#### Psathyromyia punctigeniculata

(Floch & Abonnenc, 1944)

7D6B40BA-C75B-5E5A-8F77-E9E50F63ED5B

https://sandfliesdb.herokuapp.com/

##### Distribution

Cacaulândia, Itapuã do Oeste, Monte Negro, Porto Velho, Vale do Anari

##### Notes

Azevedo et al. 1993, Gil et al. 2003, Leão et al. 2020, Pereira Júnior et al. 2019a, Pereira Júnior et al. 2019b, Resadore et al. 2018, Teles et al. 2013

#### Psathyromyia scaffi

(Damasceno & Arouck, 1956)

15D7A8EE-A383-5269-9FFA-0DE9D651D540

https://sandfliesdb.herokuapp.com/

##### Distribution

Costa Marques, Guajará-Mirim, Itapuã do Oeste, Pimenta Bueno, Porto Velho

##### Notes

Costa et al. 2021a, Galardo et al. 2015, Leão et al. 2020, Martins et al. 1965, Ogawa et al. 2016, Silva et al. 2021

#### Psychodopygus amazonensis

(Root, 1934)

FA258B9C-36A3-5BCE-833E-944E71C7AE81

https://sandfliesdb.herokuapp.com/

##### Distribution

Buritis, Cacaulândia, Campo Novo, Costa Marques, Guajará-Mirim, Itapuã do Oeste, Ji-Paraná, Monte Negro, Nova Mamoré, Porto Velho, Vale do Anari

##### Notes

Azevedo et al. 1993, Costa et al. 2021a, Galardo et al. 2015, Gil et al. 2003, Leão et al. 2020, Ogawa et al. 2016, Pereira Júnior et al. 2019a, Pereira Júnior et al. 2019b, Resadore et al. 2017, Resadore et al. 2018, Silva et al. 2021, Teles et al. 2013

#### Psychodopygus ayrozai

(Barretto & Coutinho, 1940)

37647816-F2E8-576E-8D85-1066F6264D37

https://sandfliesdb.herokuapp.com/

##### Distribution

Buritis, Cacaulândia, Campo Novo, Itapuã do Oeste, Ji-Paraná, Monte Negro, Nova Mamoré, Porto Velho, Vale do Anari

##### Notes

Azevedo et al. 1993, Galardo et al. 2015, Gil et al. 2003, Leão et al. 2020, Pereira Júnior et al. 2019a, Pereira Júnior et al. 2019b, Resadore et al. 2017, Silva et al. 2021, Torchitte et al. 2020

#### Psychodopygus bispinosus

(Fairchild & Hertig, 1951)

7267C95C-5806-5AD6-A5C5-933055CEE870

https://sandfliesdb.herokuapp.com/

##### Distribution

Buritis, Cacaulândia, Campo Novo, Itapuã do Oeste, Machadinho d'Oeste, Monte Negro, Nova Mamoré, Porto Velho, Vale do Anari

##### Notes

Costa et al. 2021b, Gil et al. 2003, Leão et al. 2020, Pereira Júnior et al. 2019a, Pereira Júnior et al. 2019b, Silva et al. 2021

#### Psychodopygus carrerai

(Barretto, 1946)

F859558D-C61C-50F1-9763-C54914D37E49

https://sandfliesdb.herokuapp.com/

##### Distribution

Buritis, Cacaulândia, Cacoal, Campo Novo, Candeias do Jamari, Costa Marques, Itapuã do Oeste, Ji-Paraná, Machadinho d'Oeste, Monte Negro, Nova Mamoré, Pimenta Bueno, Porto Velho, Vale do Anari

##### Notes

Azevedo et al. 1993, Costa et al. 2021a, Galardo et al. 2015, Gil et al. 2003, Grimaldi Júnior et al. 1991, Leão et al. 2020, Pereira Júnior et al. 2019a, Pereira Júnior et al. 2015, Resadore et al. 2017, Resadore et al. 2018, Silva et al. 2021, Teles et al. 2013, Torchitte et al. 2020

#### Psychodopygus chagasi

(Costa Lima, 1941)

6D51E3C3-8846-5AC4-B5AB-A63FFCFC6174

https://sandfliesdb.herokuapp.com/

##### Distribution

Cacaulândia, Cacoal, Itapuã do Oeste, Ji-Paraná, Monte Negro, Nova Mamoré, Pimenta Bueno, Porto Velho, São Francisco do Guaporé, Vale do Anari, Vilhena

##### Notes

Costa et al. 2021a, Galardo et al. 2015, Leão et al. 2020, Pereira Júnior et al. 2019a, Pereira Júnior et al. 2015, Resadore et al. 2017, Resadore et al. 2018, Torchitte et al. 2020

#### Psychodopygus claustrei

(Abonnenc, Léger & Fauran, 1979)

8EA4B332-5BAF-546C-B509-89BA38BD04F5

https://sandfliesdb.herokuapp.com/

##### Distribution

Buritis, Cacaulândia, Cacoal, Campo Novo, Guajará-Mirim, Itapuã do Oeste, Ji-Paraná, Machadinho d'Oeste, Monte Negro, Nova Mamoré, Pimenta Bueno, Porto Velho, São Francisco do Guaporé, Vale do Anari, Vilhena

##### Notes

Azevedo et al. 1993, Costa et al. 2021a, Galardo et al. 2015, Gil et al. 2003, Leão et al. 2020, Pereira Júnior et al. 2019a, Pereira Júnior et al. 2015, Resadore et al. 2017, Resadore et al. 2018, Silva et al. 2021, Teles et al. 2013, Torchitte et al. 2020

#### Psychodopygus complexus

(Mangabeira, 1941)

74CAF478-6D4B-538A-83A7-8F4CC748FEB9

https://sandfliesdb.herokuapp.com/

##### Distribution

Buritis, Cacaulândia, Campo Novo, Itapuã do Oeste, Ji-Paraná, Monte Negro, Nova Mamoré, Pimenta Bueno, Porto Velho, Vale do Anari

##### Notes

Costa et al. 2021a, Galardo et al. 2015, Gil et al. 2003, Leão et al. 2020, Pereira Júnior et al. 2019a, Pereira Júnior et al. 2015, Resadore et al. 2017, Resadore et al. 2018, Silva et al. 2021, Teles et al. 2013, Torchitte et al. 2020

#### Psychodopygus corossoniensis

(Le Pont & Pajot, 1978)

EB92D4C8-B9BE-5822-A22B-290206CA8149

https://sandfliesdb.herokuapp.com/

##### Distribution

Buritis, Cacaulândia, Campo Novo, Monte Negro, Porto Velho

##### Notes

Galardo et al. 2015, Gil et al. 2003

#### Psychodopygus davisi

(Root, 1934)

E985DCCB-F761-5921-80E8-3414B38E258F

https://sandfliesdb.herokuapp.com/

##### Distribution

Buritis, Cacaulândia, Cacoal, Campo Novo, Candeias do Jamari, Costa Marques, Guajará-Mirim, Itapuã do Oeste, Ji-Paraná, Machadinho d'Oeste, Monte Negro, Nova Mamoré, Pimenta Bueno, Porto Velho, São Francisco do Guaporé, Vale do Anari, Vilhena

##### Notes

Azevedo et al. 1993, Costa et al. 2021a, Galardo et al. 2015, Gil et al. 2003, Grimaldi Júnior et al. 1991, Leão et al. 2020, Martins et al. 1965, Pereira Júnior et al. 2019a, Pereira Júnior et al. 2015, Resadore et al. 2017, Resadore et al. 2018, Silva et al. 2021, Teles et al. 2013, Torchitte et al. 2020

#### Psychodopygus francoisleponti

Zapata, Depaquit & León 2012

2608888B-2762-5477-8C93-BF57C09F46F9

https://sandfliesdb.herokuapp.com/

##### Distribution

Porto Velho

##### Notes

Silva et al. 2022

#### Psychodopygus geniculatus

(Mangabeira,1941)

6324B87D-E329-5D4E-AB5B-119635CFE849

https://sandfliesdb.herokuapp.com/

##### Distribution

Buritis, Cacaulândia, Cacoal, Campo Novo, Itapuã do Oeste, Ji-Paraná, Machadinho d'Oeste, Monte Negro, Nova Mamoré, Porto Velho, São Francisco do Guaporé, Vale do Anari

##### Notes

Costa et al. 2021a, Galardo et al. 2015, Gil et al. 2003, Leão et al. 2020, Ogawa et al. 2016, Pereira Júnior et al. 2019a, Pereira Júnior et al. 2019b, Resadore et al. 2017, Resadore et al. 2018, Silva et al. 2021, Teles et al. 2013, Torchitte et al. 2020

#### Psychodopygus guyanensis

(Floch & Abonnenc, 1941)

DBB7AE9E-29D6-582A-9CAA-F6A39F7BBFC7

https://sandfliesdb.herokuapp.com/

##### Distribution

Ariquemes, Ji-Paraná, Porto Velho

##### Notes

Biancardi et al. 1982

#### Psychodopygus hirsutus

(Mangabeira, 1942)

13B39858-9A0E-5C71-97E1-B95FF82DB689

https://sandfliesdb.herokuapp.com/

##### Distribution

Buritis, Cacaulândia, Cacoal, Campo Novo, Costa Marques, Itapuã do Oeste, Ji-Paraná, Machadinho d'Oeste, Monte Negro, Nova Mamoré, Pimenta Bueno, Porto Velho, São Francisco do Guaporé, Vale do Anari, Vilhena

##### Notes

Costa et al. 2021a, Galardo et al. 2015, Gil et al. 2003, Leão et al. 2020, Pereira Júnior et al. 2019a, Pereira Júnior et al. 2015, Resadore et al. 2017, Resadore et al. 2018, Silva et al. 2021, Teles et al. 2013, Torchitte et al. 2020

#### Psychodopygus lainsoni

Fraiha & Ward, 1974

D2CDE555-0C1C-57F9-9D30-9829E2876CD4

https://sandfliesdb.herokuapp.com/

##### Distribution

Buritis, Cacaulândia, Campo Novo, Itapuã do Oeste, Monte Negro, Nova Mamoré, Porto Velho, Vale do Anari

##### Notes

Azevedo et al. 1993, Gil et al. 2003, Leão et al. 2020, Pereira Júnior et al. 2019a, Pereira Júnior et al. 2015, Resadore et al. 2017, Resadore et al. 2018, Silva et al. 2021, Teles et al. 2013

#### Psychodopygus leonidasdeanei

Fraiha, Ryan, Ward, Lainson & Shaw, 1986

EA64067A-F836-5C3B-B459-61CD5D47DC5D

https://sandfliesdb.herokuapp.com/

##### Distribution

Itapuã do Oeste, Nova Mamoré, Porto Velho, Vale do Anari

##### Notes

Galardo et al. 2015, Leão et al. 2020, Pereira Júnior et al. 2019a, Pereira Júnior et al. 2019b, Silva et al. 2021

#### Psychodopygus llanosmartinsi

Fraiha & Ward, 1980

61432812-EA3F-5EF6-AFE8-482CABD0DB27

https://sandfliesdb.herokuapp.com/

##### Distribution

Cacaulândia, Costa Marques, Guajará-Mirim, Itapuã do Oeste, Machadinho d'Oeste, Monte Negro, Nova Mamoré, Pimenta Bueno, Porto Velho, São Francisco do Guaporé, Vale do Anari

##### Notes

Costa et al. 2021a, Leão et al. 2020, Pereira Júnior et al. 2019a, Pereira Júnior et al. 2019b, Resadore et al. 2018, Silva et al. 2021

#### Psychodopygus paraensis

(Costa Lima, 1941)

720688B6-4E6B-51C3-A814-54A608CE501B

https://sandfliesdb.herokuapp.com/

##### Distribution

Buritis, Cacaulândia, Cacoal, Campo Novo, Guajará-Mirim, Itapuã do Oeste, Monte Negro, Nova Mamoré, Porto Velho, Vale do Anari

##### Notes

Galardo et al. 2015, Pereira Júnior et al. 2019a, Pereira Júnior et al. 2019b, Resadore et al. 2017, Resadore et al. 2018, Silva et al. 2021

#### Psychodopygus squamiventris

(Lutz & Neiva, 1912)

5AC22451-E1D6-514D-AC14-9CF2A69A7D1C

https://sandfliesdb.herokuapp.com/

##### Distribution

Porto Velho

##### Notes

Galardo et al. 2015

#### Psychodopygus yucumensis

(Le Pont, Caillard, Tibayrenc & Desjeux, 1986)

4D045A14-79EF-5AF1-980F-5157778828ED

https://sandfliesdb.herokuapp.com/

##### Distribution

Cacaulândia, Itapuã do Oeste, Monte Negro, Nova Mamoré, Porto Velho

##### Notes

Pereira Júnior et al. 2019a, Pereira Júnior et al. 2019b, Resadore et al. 2017, Silva et al. 2021

#### Sciopemyia fluviatilis

(Floch & Abonnenc, 1944)

C8B3E68C-ACF8-56B6-A7D3-62320CE3949E

https://sandfliesdb.herokuapp.com/

##### Distribution

Costa Marques, Itapuã do Oeste, Monte Negro, Porto Velho, Vale do Anari

##### Notes

Costa et al. 2021a, Galardo et al. 2015, Leão et al. 2020, Pereira Júnior et al. 2019a, Pereira Júnior et al. 2019b, Resadore et al. 2018

#### Sciopemyia servulolimai

(Damasceno & Causey, 1945)

EAB094B5-EEB1-5B7A-AC86-CCF0DBE8C12F

https://sandfliesdb.herokuapp.com/

##### Distribution

Buritis, Cacaulândia, Campo Novo, Guajará-Mirim, Itapuã do Oeste, Ji-Paraná, Machadinho d'Oeste, Monte Negro, Nova Mamoré, Pimenta Bueno, Porto Velho, Vale do Anari

##### Notes

Costa et al. 2021a, Gil et al. 2003, Leão et al. 2020, Martins et al. 1965, Ogawa et al. 2016, Pereira Júnior et al. 2019a, Pereira Júnior et al. 2019b, Resadore et al. 2017, Resadore et al. 2018, Silva et al. 2021, Teles et al. 2013, Torchitte et al. 2020

#### Sciopemyia sordellii

(Shannon & Del Ponte,1927)

6F078D69-865F-53A3-9300-11307A82EDEA

https://sandfliesdb.herokuapp.com/

##### Distribution

Buritis Cacaulândia Cacoal, Campo Novo, Costa Marques, Guajará-Mirim, Itapuã do Oeste, Ji-Paraná, Machadinho d'Oeste, Monte Negro, Nova Mamoré, Pimenta Bueno, Porto Velho, São Francisco do Guaporé, Vale do Anari

##### Notes

Costa et al. 2021a, Galardo et al. 2015, Gil et al. 2003, Leão et al. 2020, Martins et al. 1965, Ogawa et al. 2016, Pereira Júnior et al. 2019a, Pereira Júnior et al. 2019b, Resadore et al. 2017, Resadore et al. 2018, Silva et al. 2021, Teles et al. 2013, Torchitte et al. 2020

#### Sciopemyia vattierae

(Le Pont & Desjeux, 1992)

07A8BD8B-3244-51CD-B966-309CA6052097

https://sandfliesdb.herokuapp.com/

##### Distribution

Costa Marques, Machadinho d'Oeste, Pimenta Bueno, Porto Velho, São Francisco do Guaporé

##### Notes

Costa et al. 2021a, Silva et al. 2021

#### Trichophoromyia auraensis

(Mangabeira, 1942)

446DBCA5-76F3-5FDB-814F-B691006C6816

https://sandfliesdb.herokuapp.com/

##### Distribution

Buritis, Cacaulândia, Cacoal, Campo Novo, Guajará-Mirim, Itapuã do Oeste, Ji-Paraná, Monte Negro, Nova Mamoré, Pimenta Bueno, Porto Velho

##### Notes

Gil et al. 2003, Leão et al. 2020, Martins et al. 1965, Ogawa et al. 2016, Pereira Júnior et al. 2019a, Pereira Júnior et al. 2019b, Resadore et al. 2018, Silva et al. 2021, Teles et al. 2013

#### Trichophoromyia brachypyga

(Mangabeira, 1942)

34204F95-9DD0-507B-B4FE-8A42529227C9

https://sandfliesdb.herokuapp.com/

##### Distribution

Buritis, Cacaulândia, Campo Novo, Monte Negro, Porto Velho

##### Notes

Galardo et al. 2015, Gil et al. 2003, Resadore et al. 2017

#### Trichophoromyia castanheirai

(Damasceno, Causey & Arouck, 1945)

4C72FFD0-A690-5A75-A300-B17DABE9C865

https://sandfliesdb.herokuapp.com/

##### Distribution

Porto Velho

##### Notes

Galardo et al. 2015

#### Trichophoromyia clitella

(Young & Pérez, 1994)

60AC51DA-D065-52A1-A9ED-E4C5F03D5A11

https://sandfliesdb.herokuapp.com/

##### Distribution

Cacaulândia, Costa Marques, Guajará-Mirim, Itapuã do Oeste, Ji-Paraná, Machadinho d'Oeste, Monte Negro, Nova Mamoré, Porto Velho, Vale do Anari, Vilhena

##### Notes

Costa et al. 2021a, Leão et al. 2020, Pereira Júnior et al. 2019a, Pereira Júnior et al. 2019b, Silva et al. 2021, Teles et al. 2013, Torchitte et al. 2020

#### Trichophoromyia eurypyga

(Martins, Falcão & Silva, 1963)

DBC8048A-61C8-5272-AD7E-5ABE07382992

https://sandfliesdb.herokuapp.com/

##### Distribution

Porto Velho

##### Notes

Galardo et al. 2015

#### Trichophoromyia flochi

(Abonnenc & Chassignet, 1948)

592B5144-58FF-585E-B2D4-1B699AE67E7B

https://sandfliesdb.herokuapp.com/

##### Distribution

Guajará-Mirim, Itapuã do Oeste, Nova Mamoré, Pimenta Bueno, Porto Velho, Vale do Anari, Vilhena

##### Notes

Costa et al. 2021a, Leão et al. 2020, Martins et al. 1965, Pereira Júnior et al. 2019a, Pereira Júnior et al. 2019b, Resadore et al. 2017, Silva et al. 2021

#### Trichophoromyia ininii

(Floch & Abonnenc, 1943)

DAEE1F83-FD02-5A30-83A8-1F865EB09A50

https://sandfliesdb.herokuapp.com/

##### Distribution

Guajará-Mirim

##### Notes

speciesLink 2022

#### Trichophoromyia loretonensis

(Llanos, 1964)

0BC5A18D-3D66-5DAA-932D-DBF313459C4F

https://sandfliesdb.herokuapp.com/

##### Distribution

Itapuã do Oeste

##### Notes

Leão et al. 2020

#### Trichophoromyia melloi

(Causey & Damasceno, 1945)

9AE6570C-ACD5-5868-939B-062DC747F4FA

https://sandfliesdb.herokuapp.com/

##### Distribution

Monte Negro, Porto Velho

##### Notes

Resadore et al. 2017, Silva et al. 2021, Teles et al. 2013

#### Trichophoromyia octavioi

(Vargas, 1949)

AB3542B8-7BC5-520C-983E-78AF31508277

https://sandfliesdb.herokuapp.com/

##### Distribution

Monte Negro, Pimenta Bueno, Porto Velho

##### Notes

Ogawa et al. 2016, Resadore et al. 2017, Silva et al. 2021, Teles et al. 2013

#### Trichophoromyia readyi

(Ryan, 1986)

EED8B15D-72D8-5043-B4F0-EDDE8F055FEB

https://sandfliesdb.herokuapp.com/

##### Distribution

Itapuã do Oeste, Nova Mamoré, Porto Velho

##### Notes

Galardo et al. 2015, Pereira Júnior et al. 2019a, Pereira Júnior et al. 2019b

#### Trichophoromyia ruii

(Arias & Young, 1982)

F7965B06-EFCC-5398-BCC0-E096166EAF8C

https://sandfliesdb.herokuapp.com/

##### Distribution

Porto Velho

##### Notes

Galardo et al. 2015

#### Trichophoromyia ubiquitalis

(Mangabeira, 1942)

0D85BF0B-3A6A-52A7-9DEF-FFCFC2A79088

https://sandfliesdb.herokuapp.com/

##### Distribution

Buritis, Cacaulândia, Cacoal, Campo Novo, Costa Marques, Guajará-Mirim, Itapuã do Oeste, Ji-Paraná, Machadinho d'Oeste, Monte Negro, Nova Mamoré, Pimenta Bueno, Porto Velho, Vale do Anari, Vilhena

##### Notes

Costa et al. 2021a, Galardo et al. 2015, Gil et al. 2003, Leão et al. 2020, Martins et al. 1965, Ogawa et al. 2016, Pereira Júnior et al. 2019a, Pereira Júnior et al. 2019b, Resadore et al. 2017, Resadore et al. 2018, Silva et al. 2021, Teles et al. 2013

#### Trichopygomyia dasypodogeton

(Castro, 1939)

A3CB4590-B5FF-5546-8D44-5B1753B3EA97

https://sandfliesdb.herokuapp.com/

##### Distribution

Cacaulândia, Itapuã do Oeste, Nova Mamoré, Pimenta Bueno, Porto Velho, Vale do Anari

##### Notes

Costa et al. 2021a, Galardo et al. 2015, Gil et al. 2003, Leão et al. 2020, Pereira Júnior et al. 2019a, Pereira Júnior et al. 2019b, Resadore et al. 2018, Silva et al. 2021

#### Trichopygomyia longispina

(Mangabeira, 1942)

9A314B45-3FD2-5771-B0C4-4EDFECD7A51F

https://sandfliesdb.herokuapp.com/

##### Distribution

Cacaulândia, Itapuã do Oeste

##### Notes

Gil et al. 2003, Leão et al. 2020

#### Trichopygomyia rondoniensis

(Martins, Falcão & Silva, 1965)

484597B1-FBBE-5864-BC41-668D6BDBCF3F

https://sandfliesdb.herokuapp.com/

##### Distribution

Guajará-Mirim, Itapuã do Oeste, Porto Velho, Vilhena

##### Notes

Leão et al. 2020, Martins et al. 1965, Pereira Júnior et al. 2019a, Pereira Júnior et al. 2019b, Resadore et al. 2017, Resadore et al. 2018, Silva et al. 2021

#### Trichopygomyia trichopyga

(Floch & Abonnenc, 1945)

517A03A5-EFE8-51E3-9A96-DD4E6103CAA5

https://sandfliesdb.herokuapp.com/

##### Distribution

Cacaulândia, Porto Velho

##### Notes

Galardo et al. 2015, Gil et al. 2003

#### Trichopygomyia wagleyi

(Causey & Damasceno, 1945)

B054D87E-50F0-5E47-8B40-77AFE4A256F2

https://sandfliesdb.herokuapp.com/

##### Distribution

Itapuã do Oeste

##### Notes

Leão et al. 2020, Resadore et al. 2018

#### Viannamyia furcata

(Mangabeira, 1941)

830381B2-BB67-5A4F-93FD-AF271CAEFA9F

https://sandfliesdb.herokuapp.com/

##### Distribution

Buritis, Cacaulândia, Cacoal, Campo Novo, Guajará-Mirim, Itapuã do Oeste, Monte Negro, Nova Mamoré, Pimenta Bueno, Porto Velho, Vale do Anari, Vilhena

##### Notes

Costa et al. 2021a, Galardo et al. 2015, Gil et al. 2003, Leão et al. 2020, Martins et al. 1965, Pereira Júnior et al. 2019a, Pereira Júnior et al. 2019b, Resadore et al. 2017, Resadore et al. 2018, Silva et al. 2021, Teles et al. 2013

#### Viannamyia tuberculata

(Mangabeira, 1941)

4C658B3A-12A0-5F00-82CF-5B31708BF04A

https://sandfliesdb.herokuapp.com/

##### Distribution

Itapuã do Oeste, Nova Mamoré, Porto Velho, Vale do Anari, Vilhena

##### Notes

Galardo et al. 2015, Leão et al. 2020, Pereira Júnior et al. 2019a, Pereira Júnior et al. 2019b, Resadore et al. 2017, Resadore et al. 2018, Silva et al. 2021

## Analysis

A total of 153,155 records of sandflies captured in Rondônia were identified between 1965 and 2021, of which 147,258 were suitable for mapping (species link: 3,408, studies conducted in Rondônia: 143,850). In all, 5,887 reports were discarded mainly due to a lack of geographic coordinates. The remaining 147,258 records were distributed into four subtribes, 15 genera and 140 sand fly species. The subtribe with the most records was Psychodopygina Galati, 1995 with 132,138 records and 68 species, followed by Lutzomyiina Abonnenc & Leger, 1976 (12,534 records – 54 species), Sergentomyiina Galati, 2003 (2,296 records - 11 species) and Brumptomyiina Galati, 2003 (290 records - 7 species) (Table 1).

**Figure 1.**
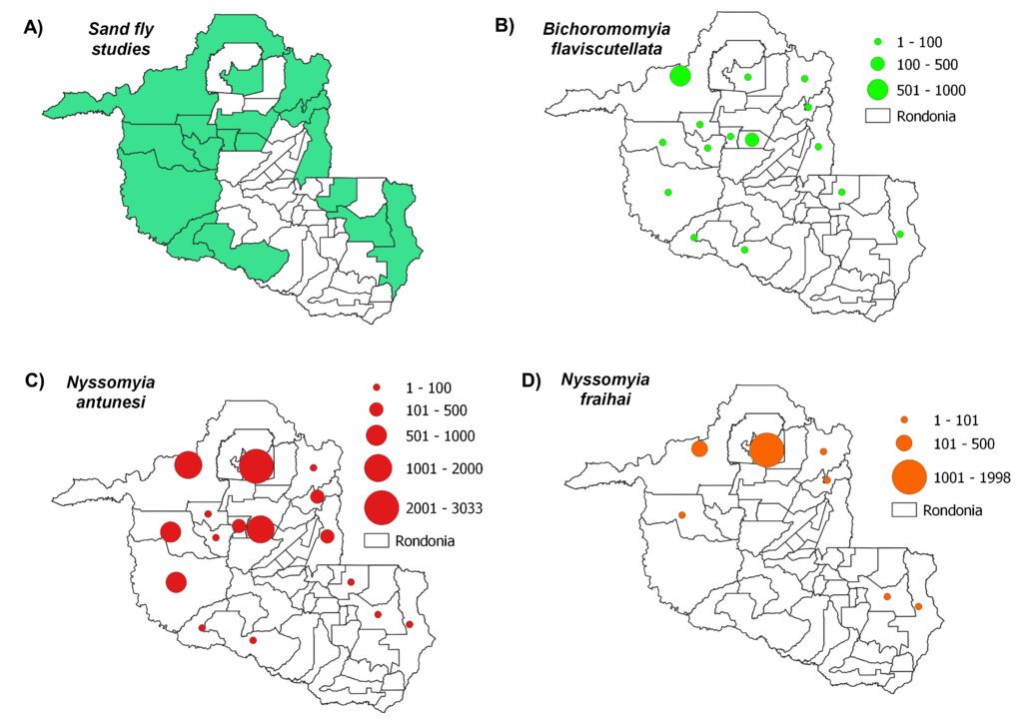
Distribution maps of sand fly species with high abundance in the State of Rondônia (Brazil). **A**) Municipalities (green) in which sandflies were reported; **B**) *Bichromomyiaflaviscutellata*; **C**) *Nyssomyiaantunesi*; **D**) *Nyssomyiafraihai*.

**Figure 2.**
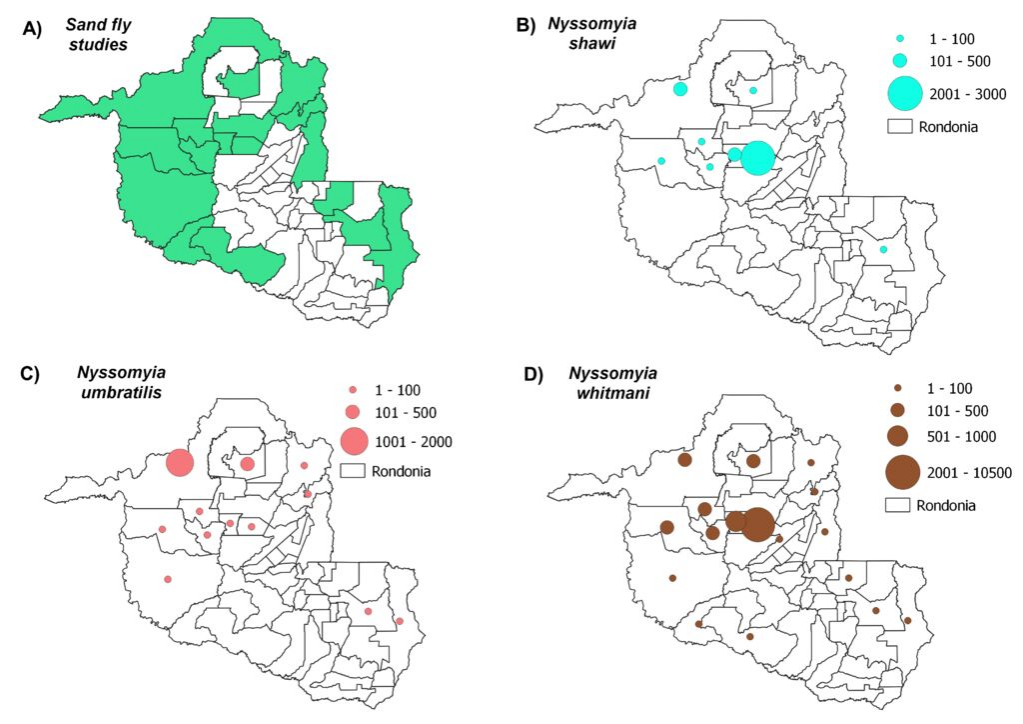
Distribution maps of sand fly species with high abundance in the State of Rondônia (Brazil). **A**) Municipalities (green) in which sandflies were reported; **B**) *Nyssomyiashawi*; **C**) Nyssomyia *umbratilis*; **D**) *Nyssomyiawhitmani*.

**Figure 3.**
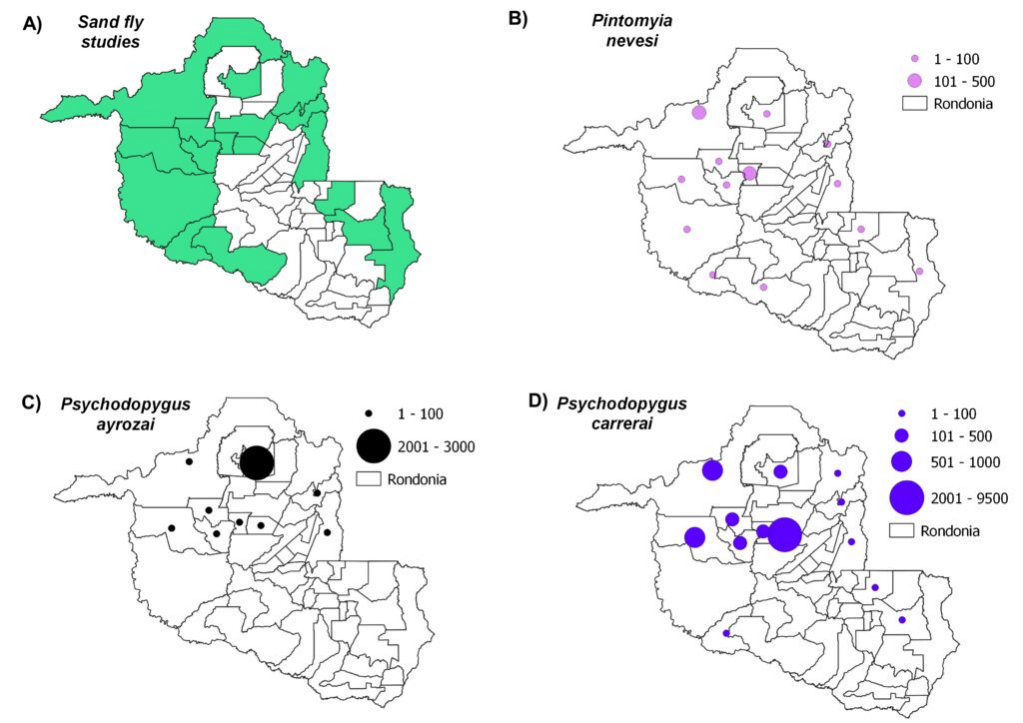
Distribution maps of sand fly species with high abundance in the State of Rondônia (Brazil). **A**) Municipalities (green) in which sandflies were reported; **B**) *Pintomyianevesi*; **C**) *Psychodopygusayrozai*; **D**) *Psychodopyguscarrerai*.

**Figure 4.**
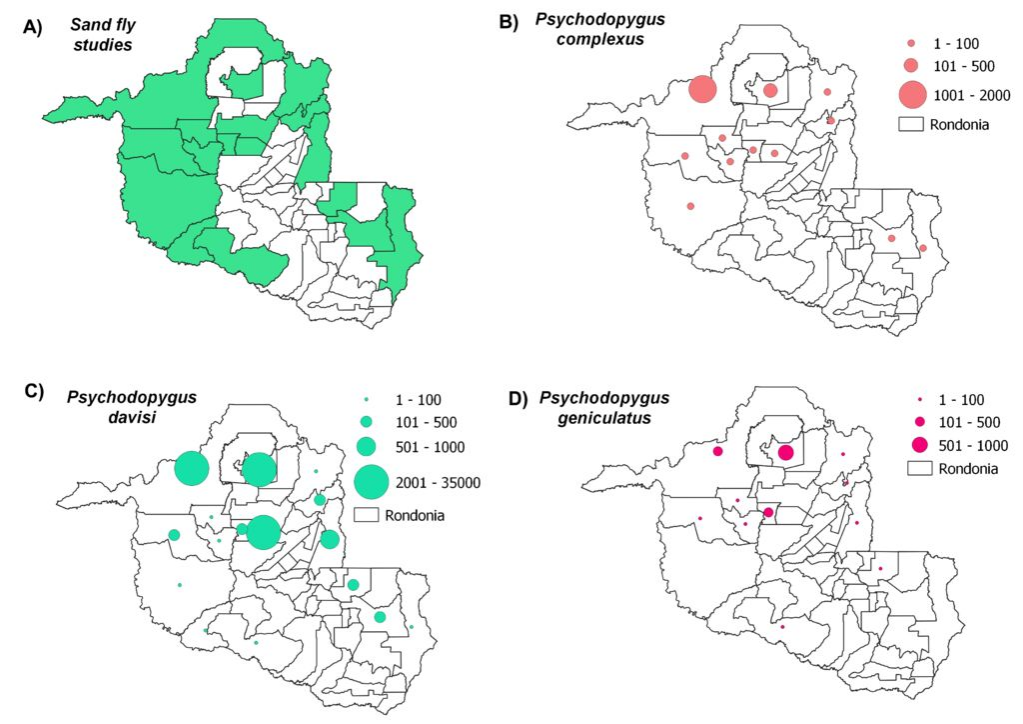
Distribution maps of sand fly species with high abundance in the State of Rondônia (Brazil). **A**) Municipalities (green) in which sandflies were reported; **B**) *Psychodopyguscomplexus*; **C**) *Psychodopygusdavisi*; **D**) *Psychodopygusgeniculatus*.

**Figure 5.**
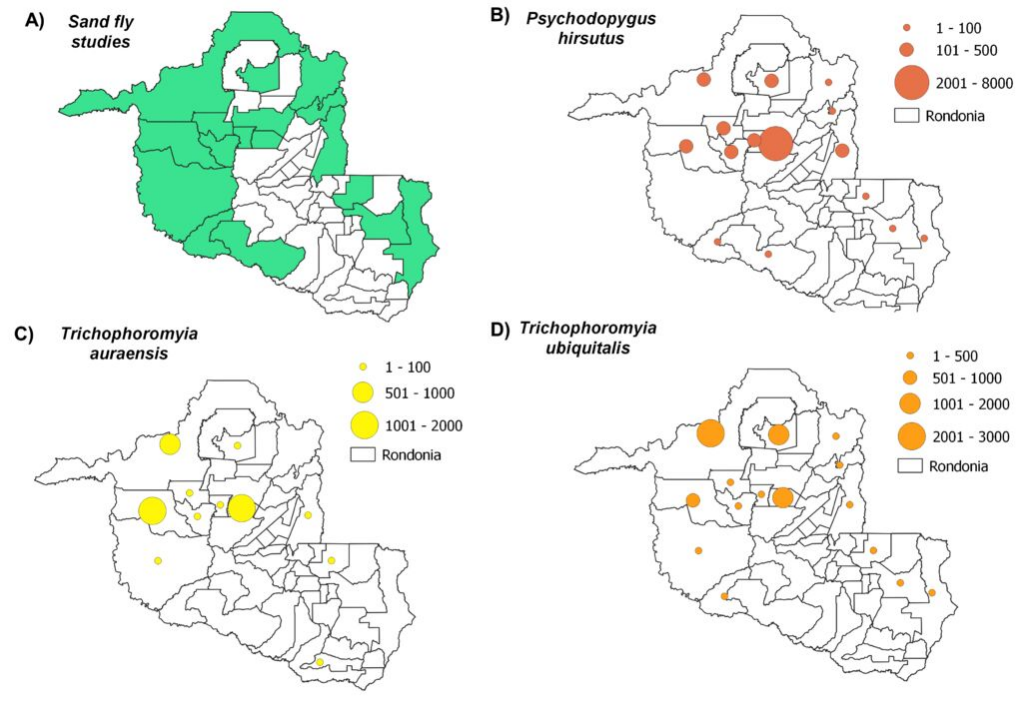
Distribution maps of sand fly species with high abundance in the State of Rondônia (Brazil). **A**) Municipalities (green) in which sandflies were reported; **B**) *Psychodopygushirsutus*; **C**) *Trichophoromyiaauraensis*; **D**) *Trichophoromyiaubiquitalis*.

Sand fly studies were conducted in 17 of the State’s 52 municipalities, with Porto Velho being the municipality with the most collection events. Considering all of the evaluated studies, the most abundant species observed were: *Psychodopygusdavisi* (Root, 1934) (43,818 records), *Nyssomyiawhitmani* (Antunes & Coutinho, 1939) (12,594), *Psychodopyguscarrerai* (Barretto, 1946) (11,840), *Psychodopygushirsutus* (Mangabeira, 1942) (9,676), *Nyssomyiaantunesi* (Coutinho, 1939) (8,847), *Trichophoromyiaubiquitalis* (Mangabeira, 1942) (5,505), *Psychodopygusgeniculatus* (Mangabeira, 1941) (4,644), *Pintomyianevesi* (Damasceno & Arouck, 1956) (4,140), *Trichophoromyiaauraensis* (Mangabeira, 1942) (3,579), *Nyssomyiashawi* (Ward, Fraiha & Ready, 1981) (3400), *Psychodopyguscomplexus* (Mangabeira, 1941) (2,659), *Nyssomyiafraihai* (Martins, Falcão & Silva, 1979) (2,504) and *Bichromomyiaflaviscutellata* (Mangabeira, 1942) (1,418). These species were most commonly distributed amongst the municipalities as follows: *N.whitmani* (17/17), *P.davisi* and *N.antunesi* (16/17), *B.flaviscutellata*, *P.hirsutus* and *T.ubiquitalis* (15/17), *P.nevesi*, *P.carrerai* (13/17), *N.umbratilis* and *P.complexus* (12/17), *T.auraensis* (11/17), *P.ayrozai* (9/17) and *N.fraihai* (7/17) (Figs 1, 2, 3, 4, 5).

**Figure 6.**
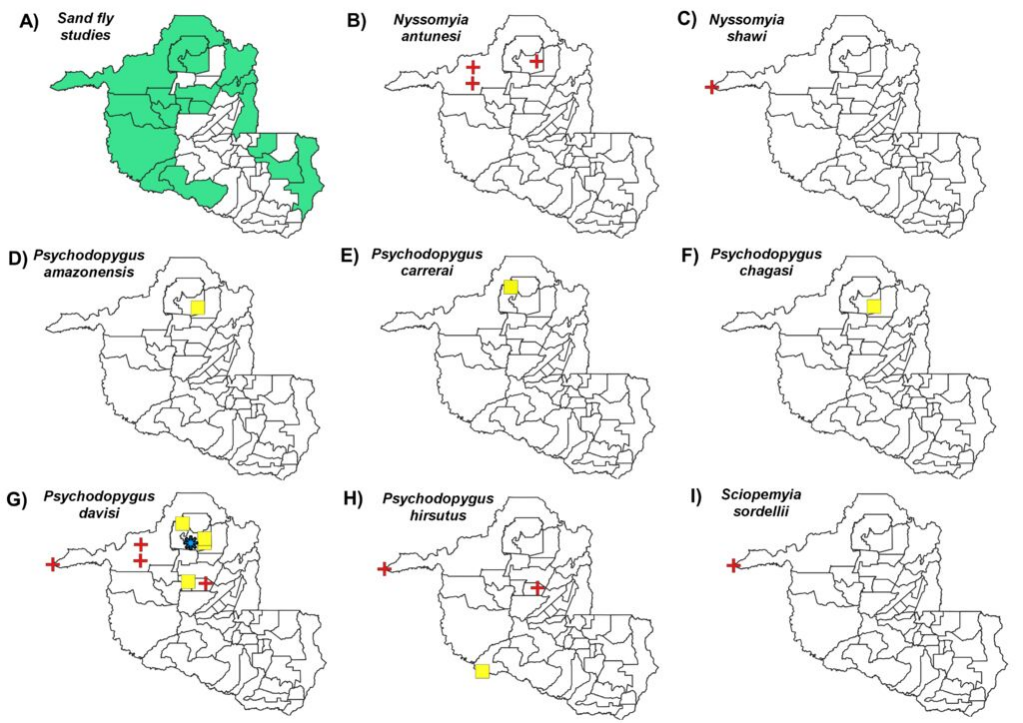
Reports of natural infection by *Leishmania* spp. in sand fly species; the blue asterisk represents *Leishmaniaamazonensis*; yellow squares represent *Leishmaniabraziliensis* and red crosses indicate *Leishmanianaiffi*. **A**) Municipalities with reports of sandflies infected with the following *Leishmania* spp.; **B**) *Nyssomyiaantunesi*; **C**) *Nyssomyiashawi*; **D**) *Psychodopygusamazonensis*; **E**) *Psychodopyguscarrerai*; **F**) *Psychodopyguschagasi*; **G**) *Psychodopygusdavisi*; **H**) *Psychodopygushirsutus*; **I**) *Sciopemyiasordellii*.

We identified 20 records of *Leishmania* infection in eight sand fly species: *N.antunesi*, *N.shawi*, *P.amazonensis*, *P.carrerai*, *P.chagasi*, *P.davisi*, *P.hirsutus* and *S.sordellii* (Fig. 6). *Leishmaniaamazonensis* (n = 1) was recorded in one species in Itapuã do Oeste. *Leishmaniabraziliensis* (n = 9) was detected in five sand fly species, reported in Costa Marques, Candeias do Jamari, Itapuã do Oeste and Monte Negro. *Leishmanianaiffi* (n = 11) was observed in five sand fly species, reported in Cacaulândia, Itapuã do Oeste and Porto Velho.

## Discussion

This study demonstrates the high diversity and distribution of sandflies, which are found in abundance in the Amazon rainforest. Although Rondônia has experienced a decrease in vegetal cover over the last 40 years (Fearnside 2014), the State still contains many conservation areas that maintain the conditions necessary to maintain assemblages, including forest fragments in which a high diversity of sandflies can be found. Considering the possibility that continued deforestation may increase the risk for increases in CL cases or even contribute to the extinction of rare species, there is a great need to explore new collection areas, mainly those not sampled, as well as to preserve areas highly impacted by deforestation.

We observed a wide species distribution amongst the evaluated municipalities, including those with *Leishmania* vector potential. Interestingly, *L.braziliensis* has been reported as the most prevalent agent in human cases recently reported in Rondônia (Cantanhêde et al. 2015, Almeida et al. 2021). Our analysis of species distribution amongst potential vectors confirmed the prevalence of *L.braziliensis*, but also indicates that *L.naiffi* also seems to be abundant. However, relatively few studies have reported natural infection by these parasites in the State, revealing low vector infection rates (Resadore et al. 2018, Pereira Júnior et al. 2019b), which makes it difficult to understand the true distribution of *Leishmania* species in sandflies throughout Rondônia.

Considering species important for *Leishmania* transmission in the Amazon, we observed low abundance of *B.flaviscutellata* across the State’s municipalities compared to other species; one possibility for this may be that all studies conducted in Rondônia used light traps to capture sand fly fauna; however, studies have demonstrated that *B.flaviscutellata* is more effectively attracted to and captured by animal rodent bait than light (Lainson and Shaw 1968, Dorval et al. 2010). If this holds true for a representative sample of this species, more specific studies implementing an active search in other environments are necessary. On the other hand, this species was identified in 15/52 of the State’s municipalities. Ecological niche models have demonstrated that *B.flaviscutellata* is present in areas where the annual mean temperature ranges from 21 to 27.6°C and annual precipitation varies between 1,139 and 3,843 mm (Carvalho et al. 2015); therefore, Rondônia has suitable conditions for developing populations of *B.flaviscutellata*.

*Nyssomyiaantunesi* is a suspected vector of *L.lindenbergi* with wide distribution throughout Brazil. This species was found in 16/52 municipalities in Rondônia with differing abundance (Pereira Júnior et al. 2019b, Silva et al. 2021). *Leishmania* DNA (Ogawa et al. 2016) was reported in this species and infection with *L.naiffi* was identified by two other studies (Leão et al. 2020, Silva et al. 2021). However, it is important to note that overlapping distribution with other recently-described species, *Nyssomyiadelsionatali* and *Nyssomyiaurbinattii* (Galati and Ovallos 2012), could be occurring. It has been suggested that these three species may constitute an “Antunesi complex,” as specific identification of *N.antunesi* and *N.urbinattii* females is complicated by similar genitalia. It is, therefore, plausible that misidentification may occur in areas where both species are present, necessitating further study to clarify what characteristics can enable the correct identification of females, leading to enhanced knowledge regarding the role of these species as CL vectors. Importantly, some females, classified in this complex, were recently reported to be infected with *L.braziliensis* DNA (Costa et al. 2021a).

*Nyssomyiafraihai* is a species that deserves discussion. Recent revalidation (Godoy and Galati 2016) evaluated specimens from the State of Bahia, Colombian topotypes of *Nyssomyiayuilliyuilli* (Young & Porter, 1972), as well as specimens identified as *N.yuilliyuilli* from other areas of Brazil, Peru and Colombia. Morphometric analysis revealed that specimens from Brazil and Peru differed from those obtained in Colombia, mainly in terms of the parameres, which led the authors to revalidate *N.fraihai*. It was also concluded that the distribution of *N.fraihai* is cis-Andean, while *N.yuilliyuilli* has been found in both Andean and Trans-Andean areas, generating speculation regarding the true distribution of these species (Godoy and Galati 2016). Many studies conducted in Rondônia have reported the presence of *N.yuilliyuilli* in their checklists, some with high abundance (Azevedo et al. 1993, Gil et al. 2003, Galardo et al. 2015, Ogawa et al. 2016, Resadore et al. 2017, Resadore et al. 2018, Pereira Júnior et al. 2019b, Leão et al. 2020, Costa et al. 2021a, Silva et al. 2021), leading us to revise the identification of some specimens identified in most studies; we concluded that the species were indeed *N.fraihai*. This observation updates the distribution of *N.fraihai* in Rondônia as being present in seven municipalities and raises further questions on the distribution of *N.yuilliyuilli* in the State.

*Nyssomyiashawi* was found to be in low abundance in most studies conducted in Rondônia; however, Gil et al. (2003) recorded a high abundance of this species, which is similar to two different reports in Acre State (Araujo-Pereira et al. 2021, Brilhante et al. 2021), which observed that this species is amongst the most frequent sand fly species predominantly occurring in primary forest environments. This species could be involved in the maintenance cycle of trypanosomatids, including *Leishmania*. Since *N.shawi* was found to be infected with *L.braziliensis* and *L.guyanensis* (Garcia et al. 2007, Bustamante et al. 2012), it is possible that populations of this species may participate in the transmission of *Leishmania* species. Although *N.shawi* was observed to be infected with *Leishmania* spp. (Ogawa et al. 2016) and *L.naiffi* (Silva et al. 2021) in Rondônia, in the State of Acre, it was observed to harbour trypanosomatids in its mid- and posterior gut (Brilhante et al. 2021); nonetheless, its vector role is still undefined.

*Nyssomyiawhitmani*, a species found in abundance mainly in the central region of the State, was reported in entomological surveys carried out in the Municipalities of Ariquemes, Cacaulândia and Monte Negro (Biancardi et al. 1982, Gil et al. 2003, Gil et al. 2009, Teles et al. 2013). This species was recorded in both forest environments and in areas that have suffered human intervention (Teles et al. 2013, Teles et al. 2016). While this species is involved in the transmission of *L.braziliensis* in some Brazilian States, there are no reports of infection by *N.whitmani* in Rondônia (Arias et al. 1985, Teles et al. 2013, Pereira Júnior et al. 2019b, Leão et al. 2020, Costa et al. 2021a, Silva et al. 2021).

*Psychodopygusayrozai* was distributed across nine municipalities, with low abundance observed in eight; higher abundance was reported in the Municipality of Itapuã do Oeste. This species is cited as a highly anthropophilic sand fly species in south-eastern Brazil, with preferential feeding activity at ground level starting at dusk, extending from approximately 1700 h - 2400 h (Aguiar and Soucasaux 1984). *Leishmanianaiffi* has been isolated and characterised from specimens of *P.ayrozai*, leading to speculation regarding this species’ participation in transmission (Arias et al. 1985).

*Psychodopyguscarrerai* and *P.complexus* are mainly reported in well-preserved forest environments (Gil et al. 2003, Resadore et al. 2018). Although both species have been found in abundance, few reports have corroborated this observation in Rondônia (Biancardi et al. 1982, Gil et al. 2003, Teles et al. 2013). *Psychodopyguscarrerai* has been identified with *L.braziliensis* in areas close to Cachoeira Samuel and Candeias do Jamari (Grimaldi Júnior et al. 1991) and this species was detected with *Leishmania* DNA in the Municipality of Itapuã do Oeste (Resadore et al. 2018). *Psychodopyguscomplexus* has only been linked to *L.braziliensis* transmission in the north-eastern State of Pará (Souza et al. 1996).

*Psychodopygushirsutus* is widely distributed across seven South American countries (Colombia, Suriname, French Guiana, Ecuador, Peru, Bolivia and Brazil) (Galati 2021). This sand fly species is present in all Brazilian States, with the exception of those in the south (Galati 2021). While broad distribution of *P.hirsutus* is notable in Rondônia, few studies have reported this species in abundance (Torchitte et al. 2020, Silva et al. 2021). Females were observed to be infected with promastigotes identified as *L.naiffi* in the State’s central region (Gil et al. 2003), as well in some rural locations of Porto Velho, where the DNA of this *Leishmania* species was detected (Silva et al. 2021); however, another study reported females infected with *L.braziliensis* DNA (Costa et al. 2021a), reinforcing the possibility of the vector role of this sand fly species in the maintenance of *Leishmania*.

*Psychodopygusdavisi* is the species most widely distributed throughout Rondônia and most studies have reported this species in abundance—our database shows this species as present in 16 municipalities. We identified many studies detecting *Leishmania* infection, with this species probably being the main vector of this protozoan in the State. Natural infection was detected by PCR from females collected in the Municipality of Monte Negro, identified through sequencing as *L.braziliensis* (Pereira Júnior et al. 2019b). *Psychodopygusdavisi* females were visualised with promastigotes identified as *L.braziliensis* (Grimaldi Júnior et al. 1991) and *L.naiffi* (Gil et al. 2003) in the State’s central region and this species was also detected together with *L.naiffi* in Porto Velho (Silva et al. 2021). Other females were recently identified with DNA from *Leishmaniaamazonensis* by PCR (Resadore et al. 2018). This species has already been detected with *Leishmania* DNA in other locations in the State of Amazonas: Lábrea (Silva et al. 2014) and Tefé (Pereira Júnior et al. 2015). *Psychodopygusdavisi* specimens were found to harbour DNA from *L.braziliensis* in Rio Branco, Acre (Ávila et al. 2018) and DNA from *L.guyanensis* in Assis Brasil, Acre, while other samples, classified as either *L.guyanensis* or *L.braziliensis* (Teles et al. 2016) have been found.

Although *Pintomyianevesi* was found to be well-distributed in Rondônia and present in 13 municipalities, no studies reported the detection of *Leishmania* DNA (Gil et al. 2003, Pereira Júnior et al. 2019b, Leão et al. 2020, Costa et al. 2021a, Silva et al. 2021). Nonetheless, this species was detected with *L.braziliensis* DNA in Acre, suggesting the vector potential of this species (Ávila et al. 2018).

*Trichophoromyiaauraensis* and *T.ubiquitalis* are likely *Leishmania* vectors in Rondônia (Santos and Silveira 2020). *Trichophoromyiaubiquitalis* has already been detected with *Leishmania* DNA in the Municipality of Porto Velho (Ogawa et al. 2016, Resadore et al. 2017). This species was observed to be infected with *Leishmania* DNA in the neighbouring areas of Lábrea, Amazonas (Silva et al. 2014) and with *Leishmanialainsoni* DNA in the Municipality of Tefé, Amazonas (Pereira Júnior et al. 2015). *Trichophoromyiaauraensis* has been reported in abundance in the Municipality of Guajará-Mirim and in municipalities located in the central region of the State (Buritis, Cacaulândia, Campo Novo and Monte Negro), as well as in Porto Velho (Biancardi et al. 1982, Gil et al. 2003, Ogawa et al. 2016, Pereira Júnior et al. 2019a). In addition, some specimens were detected with *Leishmania* DNA in Porto Velho (Ogawa et al. 2016) and Itapuã do Oeste (Resadore et al. 2018). Silva et al. (2021) observed *Trichophoromyia* spp. females infected with *L.naiffi*, which could not be identified on a species level due to morphological similarity; however, due to the collection of *T.auraensis* males, the authors suggested the possibility of some females also being *T.auraensis*. This species has also been considered abundant in studies carried out in the State of Acre, with presence reported in both forest and peri-domiciliary environments (Teles et al. 2016, Araujo-Pereira et al. 2017, Ávila et al. 2018) and *Leishmania* DNA documented in the Municipalities of Assis Brasil (Teles et al. 2016) and Rio Branco/AC (Araujo-Pereira et al. 2017).

Amongst all reports of species identified in Rondônia, we expostulate that some were incorrectly classified in the State: *Bichromomyiainornata*, *Evandromyia sp. de Baduel*, *Micropygomyiacayennensiscayennensis* (Floch & Abonnenc, 1941), *N.yuilliyuilli* (already cited), *Pintomyiaodax* (Fairchild & Hertig, 1961), *Psathyromyiarunoides* and *Psathyromyiashannoni*. However, further taxonomic study is required to definitively confirm this speculation.

The original description of *Bichromomyiainornata*, by Martins et al. (1965), is the only report of this species in the State. Distribution has been reported in Bolivia, as well as in the Brazilian States of Amazonas, Rondônia and Maranhão (Galati 2021). The species was originally described as having a dark scutellum, the same colour as the mesonotum; however, when examining the holotype, Galati characterised this structure as transparent, differently from the mesonotum, which was reportedly indistinguishable from *B.flaviscutellata* (Galati 2021). While the possibility exists that the species found in Rondônia is indeed *B.flaviscutellata*, further study is needed to confirm the taxonomic status of this species.

*Evandromyia sp. de Baduel* has only been recorded in two studies (Martins et al. 1965, Biancardi et al. 1982). Reports in Rondônia may have confused it with other species, such as *Evandromyiaandersoni* males or *Evandromyiabacula* females (Galati 2021). Although a study reported the occurrence of *Micropygomyiacayennensiscayennensis* in the State (Castellón Bermúdez 2009), this was likely incorrectly identified, as the original description cited (Gil et al. 2003) contains no reference to this species. The original report of *Pintomyiaodax* (Biancardi et al. 1982) is probably a misidentification of *Pintomyiafiocruzi* (Pereira Júnior et al. 2019a), as the latter species was not described when *P.odax* was first reported in the State. *Psathyromyiarunoides* has been cited in seven studies in Rondônia (Martins et al. 1965, Biancardi et al. 1982, Gil et al. 2003, Resadore et al. 2017, Pereira Júnior et al. 2019b, Torchitte et al. 2020). A later study that evaluated morphological and molecular evidence suggested that all specimens, in fact, belonged to *Psathyromyiapradobarrientosi*, a species very similarly to *P.runoides*, leading these authors to speculate that *P.runoides* is not distributed in Rondônia (Costa et al. 2021b).

*Psathyromyiashannoni* is reportedly widely distributed throughout the Americas, yet this has been refuted by evidence of misidentification (Sábio et al. 2014). While some studies in Rondônia have reported the identification of this species (Martins et al. 1965, Biancardi et al. 1982, Azevedo et al. 1993, Gil et al. 2003, Gil et al. 2009), it is likely that these reports could have corresponded to *Psathyromyiabigeniculata*, a species of the Shannoni series that has been identified by our group (Pereira Júnior et al. 2019b, Leão et al. 2020, Costa et al. 2021b). Molecular studies suggest the possibility that this species belongs to a cryptic species complex (Florin et al. 2011).

Other species require further taxonomic study due to the possibility of misidentification arising from morphological discrepancies, such as members of Guyanensis series: *Psychodopyguscorossoniensis*, *Psychodopygusguyanensis* and *Psychodopygusgeniculatus*. Several populations of this series collected for molecular and morphological study in Ecuador resulted in the identification of *P.geniculatus*, *Psychodopygusluisleoni* Leon, Mollinedo & Le Pont, 2009 and *P.corossoniensis.* Two populations were observed within *P.geniculatus* and one was described as a new species, *Psychodopygusfrancoisleponti* (Zapata et al. 2012). Although this species was recently recorded in Rondônia (Silva et al. 2022), our group identified some differences when analysing specimens classified as *P.geniculatus* (Resadore et al. 2018, Pereira Júnior et al. 2019b, Torchitte et al. 2020). We, therefore, suggest that future taxonomic studies clarify whether these specimens are, indeed, correctly classified, since a study conducted in Ecuador resulted in the reclassification of specimens in the Guyanensis series (Zapata et al. 2012).

*Lutzomyialongipalpis*, the main vector of *Leishmaniainfantum* Nicolle, 1908, has rarely been reported in Rondônia, which could be explained by some factors. Many studies have demonstrated that the sylvatic distribution of this species could be restricted in accordance with its populational structure. Moreover, many studies have shown that light traps are not considered an ideal method for capturing this species, necessitating the addition of pheromones or active search to obtain higher numbers of specimens (González et al. 2020). Nevertheless, this species remains important, as cases of VL in humans tend to follow reports of canine VL in Rondônia.

The information evaluated in our study permitted a wide-ranging review of accumulated reports on sand flies over many years, thus enabling the construction of a robust database with information on these insects in the State of Rondônia. Our study was limited by the exclusion of some records from the database due to the inaccurate reporting of coordinates, which may have affected the presently-described distributions of some species. Nevertheless, we believe that the maps produced by this study could serve as reference to guide future studies investigating sand fly species in Rondônia.

## Figures and Tables

**Table 1.** Records of sandflies in Rondônia state obtained from scientific literature and *speciesLink*.

**Brumptomyiina Galati, 2003 (7 species)**	**N**	**Brumptomyiina Galati, 2003 (7 species)**	**N**
*Brumptomyiaavellari* (Costa Lima, 1932)	39	*Brumptomyiamesai* Sherlock, 1962	2
*Brumptomyiabrumpti* (Larrousse, 1920)	97	*Brumptomyiapentacantha* (Barretto, 1947)	8
*Brumptomyiacunhai* (Mangabeira, 1942)	4	*Brumptomyiapintoi* (Costa Lima, 1932)	18
		*Brumptomyiatravassosi* (Mangabeira, 1942)	122
**Lutzomyiina Abonnenc & Leger, 1976 (54 species)**	**N**	**Lutzomyiina Abonnenc & Leger, 1976 (54 species)**	**N**
*Evandromyiaandersoni* (Le Pont & Desjeux, 1988)	1	*Lutzomyiagomezi* (Nitzulescu, 1931)	459
*Evandromyiaapurinan* Shimabukuro, Figueira & Silva, 2013	12	*Lutzomyialongipalpis* (Lutz & Neiva, 1912)	47
*Evandromyiabacula* (Martins, Falcão & Silva, 1965)	307	*Lutzomyiamarinkellei* Young, 1979	7
*Evandromyiabegonae* (Ortiz & Torrez, 1975)	12	*Lutzomyiasherlocki* Martins, Falcão & Silva, 1971	729
*Evandromyiabrachyphalla* (Mangabeira, 1941)	9	*Migonemyiacerqueirai* (Causey & Damasceno, 1945)	2
*Evandromyiacarmelinoi* (Ryan, Lainson, Fraiha & Shaw, 1986)	1	*Migonemyiamigonei* (França, 1920)	331
*Evandromyiaevandroi* (Costa Lima & Antunes, 1936)	19	*Pintomyiadamascenoi* (Mangabeira, 1941)	23
*Evandromyiageorgii* Freitas & Barrett, 2002	286	*Pintomyiaduckei* Oliveira, Alencar & Freitas, 2018	8
*Evandromyiainfraspinosa* (Mangabeira, 1941)	263	*Pintomyiafiocruzi* Pereira Júnior, Pessoa, Marialva & Medeiros, 2019	34
*Evandromyiainpai* (Young & Arias, 1977)	2	*Pintomyiagruta* (Ryan, 1986)	12
*Evandromyialenti* (Mangabeira, 1938)	11	*Pintomyianevesi* (Damasceno & Arouck, 1956)	4140
*Evandromyiamonstruosa* (Floch & Abonnenc, 1944)	42	*Pintomyiaodax* (Fairchild & Hertig, 1961)	1
*Evandromyiapinottii* (Damasceno & Arouck, 1956)	4	*Pintomyiapacae* (Floch & Abonnenc, 1943)	1
*Evandromyiapiperiformis* Godoy, Cunha & Galati, 2017	21	*Pintomyiaserrana* (Damasceno & Arouck, 1949)	308
*Evandromyiasaulensis* (Floch & Abonnenc, 1944)	467	*Pressatiacalcarata* (Martins & Silva, 1964)	15
*Evandromyiasericea* (Floch & Abonnenc, 1944)	8	*Pressatiachoti* (Floch & Abonnenc, 1941)	89
*Evandromyia sp. de Baduel* (Floch & Abonnenc, 1945)	1	*Pressatiatriacantha* (Mangabeira, 1942)	984
*Evandromyiatarapacaensis* (Le Pont, Torrez-Espejo & Galati, 1997)	373	*Pressatiatrispinosa* (Mangabeira, 1942)	80
*Evandromyiatermitophila* (Martins, Falcão & Silva, 1964)	102	*Sciopemyiafluviatilis* (Flochi & Abonnenc, 1944)	69
*Evandromyiawalkeri* (Newstead, 1914)	194	*Sciopemyiaservulolimai* (Damasceno & Causey, 1945)	123
*Evandromyiawilliamsi* (Damasceno, Causey & Arouck, 1945)	22	*Sciopemyiasordellii* (Shannon & Del Ponte, 1927)	1349
*Evandromyiawilsoni* (Damasceno & Causey, 1945)	461	*Sciopemyiavattierae* (Le Pont & Desjeux, 1992)	21
*Lutzomyiacaligata* (Martins,Falcão & Silva, 1965)	2	*Trichopygomyiadasypodogeton* (Castro, 1939)	569
*Lutzomyiacarvalhoi* (Damasceno, Causey & Arouck, 1945)	23	*Trichopygomyialongispina* (Mangabeira, 1942)	4
*Lutzomyiaevangelistai* Martins & Fraiha, 1971	235	*Trichopygomyiarondoniensis* (Martins, Falcão & Silva, 1965)	121
*Lutzomyiafalcata* Young & Morales, 1994	2	*Trichopygomyiatrichopyga* (Floch & Abonnenc, 1945)	73
*Lutzomyiaflabellata* Martins & Silva, 1964	11	*Trichopygomyiawagleyi* (Causey & Damasceno, 1945)	45
**Psychodopygina Galati, 1995 (68 species)**	**N**	**Psychodopygina Galati, 1995 (68 species)**	**N**
*Bichromomyiaflaviscutellata* (Mangabeira, 1942)	1418	*Psychodopygusamazonensis* (Root, 1934)	562
*Bichromomyiainornata* (Martins, Falcão & Silva, 1965)	2	*Psychodopygusayrozai* (Barretto & Coutinho, 1940)	2308
*Bichromomyiaolmecanociva* (Young & Arias, 1982)	14	*Psychodopygusbispinosus* (Fairchild & Hertig, 1951)	352
*Bichromomyiareducta* (Feliciangeli, Ramirez Pérez & Ramirez, 1988)	15	*Psychodopyguscarrerai* (Barretto, 1946)	11840
*Martinsmyiawaltoni* (Arias, Freitas & Barrett, 1984)	28	*Psychodopyguschagasi* (Costa Lima, 1941)	1436
*Nyssomyiaanduzei* (Rozeboom, 1942)	219	*Psychodopygusclaustrei* (Abonnenc, Léger & Fauran, 1979)	1827
*Nyssomyiaantunesi* (Coutinho, 1939)	8847	*Psychodopyguscomplexus* (Mangabeira, 1941)	2659
*Nyssomyiadelsionatali* Galati & Galvis, 2012	36	*Psychodopyguscorossoniensis* (Le Pont & Desjeux, 1978)	652
*Nyssomyiafraihai* (Martins, Falcão & Silva, 1979)	2504	*Psychodopygusdavisi* (Root, 1934)	43818
*Nyssomyiarichardwardi* (Ready & Fraiha, 1981)	1241	*Psychodopygusfrancoisleponti* (Zapata, Depaquit & León, 2012)	1
*Nyssomyiashawi* (Fraiha, Ward & Ready, 1981)	3400	*Psychodopygusgeniculatus* (Mangabeira, 1941)	4644
*Nyssomyiaurbinattii* Galati & Galvis, 2012	64	*Psychodopygusguyanensis* (Floch & Abonnenc, 1941)	3
*Nyssomyiawhitmani* Antunes & Coutinho, 1939	12594	*Psychodopygushirsutus* (Mangabeira, 1942)	9676
*Nyssomyiayullipajoti* (Abonnenc, Léger & Fauran, 1979)	2	*Psychodopyguslainsoni* Fraiha & Ward, 1974	1117
*Nyssomyiayulliyuilli* (Young & Porter, 1972)	876	*Psychodopygusleonidasdeanei* Fraiha, Ryan, Ward, Lainson & Shaw, 1986	474
*Nyssomyiaumbratilis* (Ward & Fraiha, 1977)	1782	*Psychodopygusllanosmartinsi* Fraiha & Ward, 1980	428
*Psathyromyiaabonnenci* (Floch & Chassignet, 1947)	40	*Psychodopygusparaensis* (Costa Lima, 1941)	111
*Psathyromyiaabunaensis* (Martins, Falcão & Silva, 1965)	12	*Psychodopygussquamiventris* (Lutz & Neiva, 1912)	30
*Psathyromyiaaragaoi* (Costa Lima, 1932)	351	*Psychodopygusyucumensis* (Le Pont, Caillard, Tibayrenc & Desjeux, 1986)	58
*Psathyromyiabarrettoi* (Mangabeira, 1942)	41	*Trichophoromyiaauraensis* (Mangabeira, 1942)	3579
*Psathyromyiabigeniculata* (Floch & Abonnenc, 1941)	494	*Trichophoromyiabrachypyga* (Mangabeira, 1942)	57
*Psathyromyiabrasiliensis* (Costa Lima, 1932)	14	*Trichophoromyiacastanheirai* (Damasceno, Causey & Arouck, 1945)	167
*Psathyromyiacampbelli* (Damasceno, Causey & Arouck, 1945)	142	*Trichophoromyiaclitella* (Young & Pérez, 1994)	292
*Psathyromyiacoutinhoi* (Mangabeira, 1942)	13	*Trichophoromyiaeurypyga* (Martins, Falcão & Silva, 1963)	138
*Psathyromyiadasymera* (Fairchild & Hertig, 1961)	1	*Trichophoromyiaflochi* (Abonnenc & Chassigneti, 1948)	594
*Psathyromyiadendrophyla* (Mangabeira, 1942)	1903	*Trichophoromyiaininii* (Floch & Abonnenc, 1943)	9
*Psathyromyiadreisbachi* (Causey & Damasceno, 1945)	97	*Trichophoromyialoretonensis* (Llanos, 1964)	8
*Psathyromyiaelizabethdorvalae* Brilhante, Sábio & Galati, 2017	21	*Trichophoromyiamelloi* (Causey & Damasceno, 1945)	762
*Psathyromyiahermanlenti* (Martins, Falcão & Silva, 1970)	233	*Trichophoromyiaoctavioi* (Vargas, 1949)	487
*Psathyromyiainflata* (Floch & Abonnenc, 1944)	16	*Trichophoromyiareadyi* (Ryan, 1986)	29
*Psathyromyialutziana* (Costa Lima, 1932)	268	*Trichophoromyiaruii* (Arias & Young, 1982)	1
*Psathyromyiapradobarrientosi* (Le Pont, Matias, Martinez & Dujardin, 2004)	100	*Trichophoromyiaubiquitalis* (Mangabeira, 1942)	5505
*Psathyromyiapunctigeniculata* (Floch & Abonnenc, 1944)	11	*Viannamyiafurcata* (Mangabeira, 1941)	1310
*Psathyromyiascaffi* (Damasceno & Arouck, 1956)	38	*Viannamyiatuberculata* (Mangabeira, 1941)	367
**Sergentomyiina Galati, 2003 (11 species)**	**N**	**Sergentomyiina Galati, 2003 (11 species)**	**N**
*Micropygomyiaacanthopharynx* (Martins, Falcão & Silva, 1962)	679	*Micropygomyiaperesi* (Mangabeira, 1942)	3
*Micropygomyiacayennensiscayennensis* (Floch & Abonnenc, 1941)	2	*Micropygomyiapilosa* (Damasceno & Causey, 1944)	9
*Micropygomyiaechinathopharynx* Andrade Filho, Galati, Andrade & Falcão, 2004	1	*Micropygomyiarorotaensis* (Floch & Abonnenc, 1944)	160
*Micropygomyialongipennis* (Barretto, 1946)	28	*Micropygomyiatrinidadensis* (Newstead, 1922)	1133
*Micropygomyiamicropyga* (Mangabeira, 1942)	76	*Micropygomyiavillelai* (Mangabeira, 1942)	202
*Micropygomyiaoswaldoi* (Mangabeira, 1942)	3		
